# An indirect approach to identify the healthcare services for thyroid and melanoma cancer patients in Italy: Epicost-2 project

**DOI:** 10.1177/03008916251353109

**Published:** 2025-09-04

**Authors:** Sandra Mallone, Andrea Tavilla, Tania Lopez, Daniela Pierannunzio, Luigino Dal Maso, Stefano Guzzinati, Ugo Fedeli, Alberto Gagliani, Alessandra Buja, Manuel Zorzi, Mario Fusco, Federica Toffolutti, Silvia Francisci

**Affiliations:** 1 National Center for Disease Prevention and Health Promotion, National Institute of Health, Rome, Italy; 2 Cancer Epidemiology Unit, Centro di Riferimento Oncologico di Aviano, Istituto di Ricovero e Cura a Carattere Scientifico, Aviano, Italy; 3 Regional Epidemiological Service, Veneto Cancer Registry, Azienda Zero, Padova, Italy; 4 Department of Cardiac, Thoracic and Vascular Sciences and Public Health, Hygiene and Public Health, University of Padova, Padova, Italy; 5 Registro tumori ASL Napoli 3 sud, Napoli, Italy

**Keywords:** health services data, cancer registries data, thyroid cancer, melanoma, cancer phases of care

## Abstract

**Introduction:**

An indirect approach was applied to the case-study of thyroid cancer (TC) and melanoma of the skin (MS) in Italy to identify health services (HS) for cancer patients and to enable cost estimation.

**Materials and methods:**

Within the Epicost-2 project, a self-controlled crossover design analysed TC and MS 2018 prevalent cases from Italian cancer registries. Controls (1:1) were matched to cases 18-6 months prior to diagnosis; increases between cases and controls in potentially cancer-related HS claims (P⩽5%) were identified.

**Validation:**

Oncology and clinical experts validated cancer-related HS lists using statistical, clinical, and economic criteria.

**Results:**

The approach identified 202 and 333 cancer-related HS codes for TC and MS, respectively, aligned with clinical pathways.

**Discussion:**

The indirect approach reduced validation workload by 75% versus direct one.

**Conclusion:**

The approach identifies the costs of cancer care that could also be reproduced in other countries with consistent results, and the approach applied to other cancers.

## Introduction

According to GLOBOCAN 2022, the number of prevalent cancer cases 5-year after diagnosis of thyroid cancer (TC) is over 2.9 million worldwide, while the number of prevalent cases with skin melanoma (MS) is about 1.3 million.^1^The EUROCARE-6 study estimated there were 23,711,000 people alive in 2020 in Europe with a previous cancer diagnosis, irrespective of when they were diagnosed, 5.0% of the European population. As regards cases of TC/MS, the authors estimated 734,000/196,000 prevalent cases in females and 779,000/612,000 in males, respectively.^2^ In Italy in 2018, 43,000 men and 148,000 women have been estimated as living with a diagnosis of TC and about 173,000 people with a MS diagnosis, being the fifth and sixth highest prevalent among oncological diseases.^3^ Both TC and MS continue to pose a significant cost to patients and healthcare systems. The costs of TC in Italy, as far we know, have not been estimated. In the MELODY study, the health services and their costs provided to patients with advanced melanoma were compared across the UK, Italy, and France.^4^ In Europe, a recent retrospective study on multiple integrated data sources, shows that the estimated cost of thyroid cancer management in France in 2011 to 2015 was €200 million.^5^ A more recent literature on healthcare services on MS in Italy provides estimates for the direct costs of patients with stage I MS in 2017^6^, and the direct costs of MS patients diagnosed in 2015 and 2017 by time from the diagnosis.^7^

The sustainability of the cancer burden represents a major challenge worldwide for welfare systems. Health policy makers need to know what the economic burden associated with cancer is over time and space to identify effective policies to promote allocative efficiency and sustainability.

Many papers, since the 1990s, have addressed the problem of how to define and measure the cancer cost with different methods:

- observing the entire life course considering the different cost components at population level^8-11^;
- measuring specific cost components^12^;
- selecting certain phases of treatment^13^;
- selecting clinical cohorts^14^

In Italy, the Epicost project funded by the Italian Ministry of Health, developed a first attempt to devise a comprehensive method to estimate cancer costs that involves epidemiological, clinical, health-economic and statistical components. The main project objective of Epicost was to estimate the cost of breast and colon-rectum cancers, along the life course of patients, by merging the health services data (HSD) provided to these cancer patients by the Italian National Health System (INHS) and information on cancer patients collected by the Italian Cancer Registries (CR). This overall goal was achieved through the direct approach that identifies lists of Health Services (HS) related to the tumors of interest, drawn by a pool of expert clinicians and oncologists based on clinical practice and current guidelines. Details about the methodology applied to the cases of female breast cancer are documented in Busco.^15^ The direct method is particularly suitable for cancer sites with complex patterns of care, such as breast cancer. However, as pointed out in the discussion of the paper, the development of lists of cancer-related codes is time consuming.

In this paper, we propose as an alternative, the so-named indirect approach,^16^ using the data of cancer patients collected by the Italian CRs in the framework of Epicost-2 project (second edition of the Epicost project) funded by the Italian Ministry of Health. For illustrative purposes, the method has been applied to the TC and MS case study drawn from the Epicost-2 database. This was done in agreement with expert oncologists and clinicians participating in the project. Compared to the previous project, the indirect approach reverses the steps of the direct approach - from the lists built *a priori* by the experts to the identification of the services registered in the HSD. In fact, once the statistical analysis makes it possible to identify those services as cancer-related, showing a statistically significant increase of occurrences after cancer diagnosis, the final expert contribution is the validation of the results from a clinical and oncological point of view and to associate the relative costs in specific stages of the disease. The strength of using this alternative approach will be further explained in the discussion.

## Materials

### Epicost-2 project

The Epicost-2 is a population-based retrospective project aimed to estimate costs of cancer according to three phases of care that can occur during a patients’ clinical pathway: initial (12 months after cancer diagnosis); continuing (time elapsed between initial and final); final (last 12 months before death due to cancer). At the prevalence date, phases of care are mutually exclusive. It has a cross-sectional design and includes cancer patients diagnosed at age 15 and older, alive on 1 January 2018 and followed up to 31 December 2018, collected during the entire period of registration activity from three participating CRs: Veneto, Friuli Venezia Giulia with regional coverage, and Napoli covering 20% of the regional population. Patients with multiple cancers were included if the distance between the index cancer and the previous multiple is greater than five years.

### Case study design and patient data

We devised a design borrowed from nested case-control and self-controlled crossover (SCC) observational studies^17-19^ adapted to our data, to apply an indirect approach for the identification of services and their relative costs provided to Epicost-2 prevalent cases. In detail, we selected in the Epicost-2 prevalence population a sub-set of 3017 (25% of the 12,157 TC prevalent cases) and 4176 (28% of the 14,924 MS prevalent cases) cases of TC and MS respectively, diagnosed in 1 July 2015-31 December 2017 according to the International statistical classification of disease (ICD-10) topography and morphology codes ICD-10 C73 and ICD-10 C43,^20^ alive on 1 January 2018 and followed up to 31 December 2018 (see Figure 1a). This sub-set of cancer patients (exposed to cancer) was analyzed retrospectively over time to see how many/which health services they used (outcome). This type of study design is feasible thanks to the availability of HSD related to prevalent cases from four years before to one year after 31 December 2018. Cancer patients act as their own controls as follows:

- cancer patients are followed up in the 12 months period after diagnosis;
- same patients, but not yet affected by cancer, are analyzed in terms of demand for health services from 18 months to six months before diagnosis.

**Figure l.**
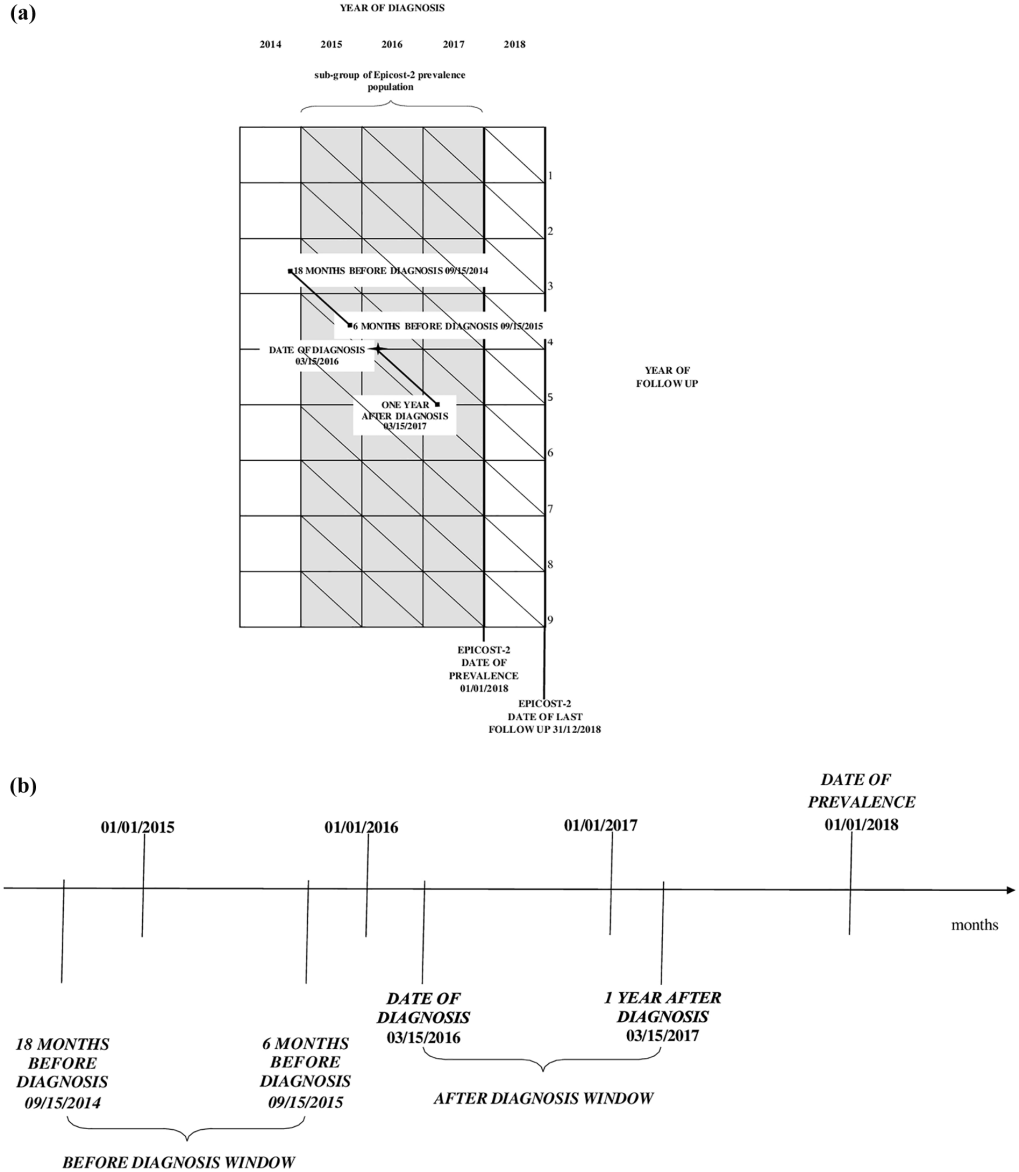
(a) Sub-set of cancer patients selected from the Epicost-2 prevalence population, with a diagnosis of thyroid cancer and melanoma of skin during the period from 1 July 2015-31 December 2017 (gray section) and followed up to 31 December 2018. Example: patient acting as his own control, diagnosed on 15 March 2016. (b) Time windows: 15 September 2014-15 September 2015 before diagnosis; 15 March 2016-15 March 2017 after diagnosis.

Since the date of diagnosis does not perfectly match the clinical course, we excluded the six months before diagnosis from the pre-diagnosis period. This choice is consistent with a previous paper^15^ on female breast cancer where the exclusion was limited to two months immediately prior to diagnosis. The exclusion period was extended to six months to reduce the chances of including procedures that occurred several months before definitive diagnosis.

We provide Figure 1b as an example: we analyzed the care pathway of a patient 1:1 matched diagnosed on 15 March 2016, whose health services occurred in the time windows 15 September 2014 – 15 September 2015 (before diagnosis) and 15 March 2016-15 March 2017 (after diagnosis).

### Source of health services data

The INHS is a public welfare system which guarantees universal healthcare in Italy. It is centrally organized under the Ministry of Health and is administered on a regional basis (19 regions and two provinces). Hospitals, clinics and ambulatories, as well as hospital/territorial pharmacies, collect their claims for services, registered it in the HSD and transmit it to the Italian regional health authority to be reimbursed. In this paper, we used the information collected in HSD, included in the Epicost-2 project, provided to cancer patients collected in Friuli Venezia Giulia, Veneto, Napoli CRs during the period 1 January 2014 -31 December 2018 and potentially linkable to the selected 3017 TC patients and 4176 of MS patients respectively. In detail, HSD include the following individual types of data: Hospital Admissions and Discharges (HA) records of discharges containing main diagnosis and procedures according to the International Classification of Diseases, 9th Revision Clinical Modification^21^; Outpatient Services (OPS) records referring to a single outpatient procedure, coded according to the International Classification of Diseases, 10th Revision Clinical Modification^21^; Drug Prescriptions (DP) and Hospital Drugs (HD) records containing information on prescribed drugs coded according to the Anatomical Therapeutic Chemical Classification System^20^ and provided by a territorial pharmacy (DP) and/or by a hospital pharmacy (HD). This last database contains high-cost drugs such as biologics, and monoclonal antibodies.^23,24^ In Italy, regional and national health authorities are legislated as collectors of personal data for surveillance purposes without explicit individual consent. Ethics committee approval for research involving this database was not required for a descriptive analysis of anonymous data without any direct or indirect intervention on patients.^25^

## Methods

We performed a linking process, deterministic, complete, one-to-many, between information in HSD and in the pooled CRs data to have generalizable estimates of the relevant cancer related codes using an anonymous identification number assigned to NHS beneficiaries. Then we conducted the statistical analysis to identify the HSD provided over time on either cases or controls. By comparing the frequencies of diagnoses/procedures/drug provided to cases and those of controls, we identified the frequencies that show a statistically significant increase after diagnosis with respect to the before-diagnosis period potentially related to cancer, i.e. cancer-related codes. In mathematical form, we define frequency of each diagnosis/procedure/drug collected in the HSD, is indicated as N*X_i_* where *i* stands for the ICD9-CM code^21^ in the HA and OPS database and for the Anatomical Therapeutic Chemical (ATC) classification system codes^22^ in the HD and DP database, which identify the services provided to cancer patients, while *X* (*X*= *B*, *A*) stands for the temporal observation window. In the HA database note that the main procedure codes are used to identify the relevant category/subcategory; when there was no procedure code, the diagnosis code is used. We compared the frequency N*A_i_* of cases in the after the diagnosis window with that of the corresponding N*B_i_* of paired controls in the before the diagnosis window. Paired frequencies (N*A_i_* ; N*B_i_*) were used to estimate the differences Δ_*i*_ between the frequency for the after/before windows Δ_*i*_= N*A_i_*-N*B_i_*= 0. A one-sided t-test procedure was applied on the positive differences Δi by code and HS flows, thus focused only on the services provided for patients after the onset of the cancer. Patients of the SCC were assigned to the relevant phase of care, according to the definition used in the Epicost study.^15,23,24^ Statistical analyses were performed using SAS version 9.4.^26^

## Validation

Once the t-test procedure estimates t and corresponding p-value for the each Δi, we built the lists of the health service by code, Δi, t-test and p, per HS flows.

A panel of expert oncologists and clinicians were asked to comment and validate these lists based on following criteria, applied in hierarchical order:

i. clinical - each health service is consistent with clinical practice and the current guidelines on the management of cancer patients.
ii. mathematical - the health services whose relative frequency is below/above 0.1% are representative of the total services.
iii. economic - the cost associated with the health services that did not appear to comply with the guidelines can be considered negligible from an economic point of view.

**Figure 2.**
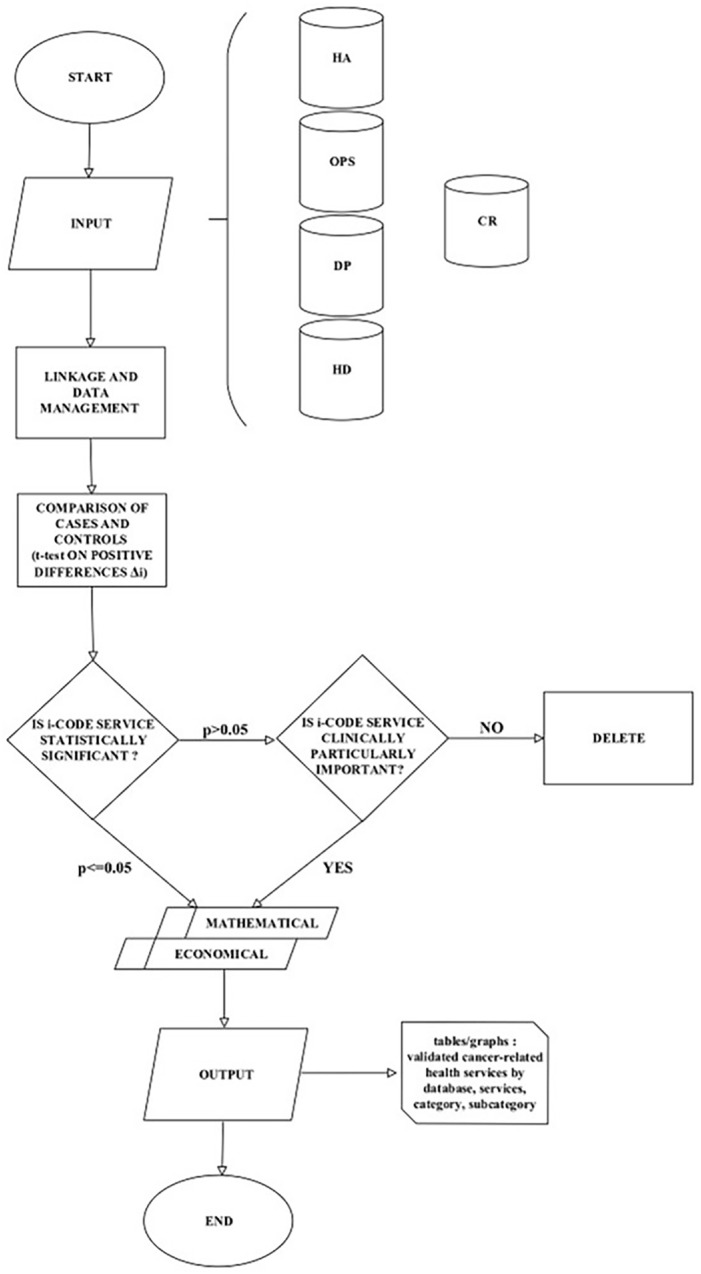
Process mapping in identifying healthcare services for cancer patients along the pathway of cancer patients using Real World Data sources: Hospital Admissions and Discharges (HA), Outpatient Services (OPS), Drug Prescriptions (DP), Hospital Drugs (HD), Cancer Registries (CR).

Figure 2 describes the process mapping in building an archive of individual records containing information on patients collected in HSD and CRs and the steps taken by the experts to arrive at a validated list of the results that emerged from the statistical analysis, using the clinical, mathematical and economic criteria described above.

Based on this fine-tuning of the list of services, the frequencies of i-codes approved by the experts were counted for the entire Epicost-2 prevalence population and classified according to the initial, continuing, and final phases of care for TC and MS respectively.

## Results

### Linked records between health services databases and cancer registries data in self-controlled crossover design

The linking process between information in HSD and cancer patients included in SCC study gave the following results for TC and MS respectively:

- 7179/8167 HA records of discharges, each record contains main diagnosis and procedures;
- 571,171/ 630,894 OPS records, each record refers to a single outpatient procedure;
- 219,951/ 293,469 DP records and 16,918/31,669 HD records, each record contains information on prescribed drugs provided by a territorial pharmacy (DP) and/or by a hospital pharmacy (HD) respectively.

### Cancer-related health services provided to TC and MS cases in the self-controlled crossover design

Table 1 shows the HSD records for patients with TC and MS by health service used in identifying the observation windows in the SCC design and in applying t-test on the differences Δ_*i*_. To explain the reading of Table 1, we provide the following example. In the case of the TC for HA database, we identified 5534 records out of a total of 7179 to be used for the comparison of before and post diagnosis windows. We estimated 895/605 differences Δ_i_ for diagnoses/procedures, of which 149/121 were statistically significant (p<0.05), but only 58/50 of these were validated by the expert panel. The lists of the cancer-related services for TC and MS in the SCC, clinically validated, are reported in Online Appendix A sorted by ICD9-CM/ATC code, with description, category/subcategory.

**Table 1.** Paired records included in the before/after diagnosis windows collected in the Health Services Database for thyroid and melanoma cancer patients, positives differences Δ_i_ (t-tested, statistically significant, clinically validated) computed by service. Self-controlled crossover design: 3017 for thyroid and 4176 for melanoma cancer patients, diagnosed 1 July 2015-31 December 2017 and followed up to 31 December 2018.

Health Services Database	Service	Thyroid Cancer				Melanoma			
		Paired records included	Positives differences Δ_i_			Paired records included	Positives differences Δ_i_		
			t-tested	statistically significant	clinically validated		t-tested	statistically significant	clinically validated
**Hospital Admissions and Discharges**	Diagnosis	5,534	895	149	58	4,979	1,068	195	63
	Procedure		605	121	50		728	166	108
**Outpatients Services**	Procedure	255,041	1,480	243	65	272,215	1,548	371	75
**Drugs Pharmacy**	Drug	87,496	474	61	12	112,910	514	80	54
**Hospital Drugs**	Drug	7,861	198	24	17	13,908	238	51	33

Note: the lists of the cancer-related services for TC and MS in the SCC, clinically validated, are reported in Online Appendix A sorted by ICD9-CM/ATC code, with description, category/subcategory.

### Health services provided to TC cases in the Epicost-2 population

For descriptive purposes, we grouped the fine-tuning diagnosis/procedures/drugs according to clinically relevant categories and/or subcategories by HSD. The 12,157 prevalent cases of TC in the Epicost-2 population according to the phase of care are: 1199 patients in the initial phase (9.9%), 10,855 in the continuing phase (89.9%) and 103 in the final one (0.3%), respectively. Table 2 shows the frequencies of health care services provided to TC prevalent cases, ordered by *i*-codes, in each phase of care. For a total of 20,465 hospitalizations, the patients received at least 10,063 main cancer-related procedures and at least 10,402 main diagnoses without corresponding procedures. In the initial (54.4%) and the continuing (49.1%) phases, grouping on procedures subcategory almost half of the surgery provided are assigned to ‘Total thyroidectomy’ and ‘Partial thyroidectomy’, while in the final phase 51.1% are assigned to ‘High diagnostic’ and ‘Support therapy’. Among ambulatory services, it stands out that ‘Blood test’ is the most typical routine component of TC monitoring in the ‘Diagnosis and monitoring’ category administered to these patients. From the initial to the final phase of treatment, the amount of ‘Thyroid hormone’ prescriptions dispensed in territorial pharmacies are 53.1% of all drugs prescribed while in hospital pharmacies the percentage does not reach 1%. ‘Support therapy’ follows as the largest quantity of prescriptions dispensed in both pharmacies.

**Table 2.** Diagnoses/procedures/drugs provided for thyroid cancer (TC) patients, collected in the: Hospital Admissions and Discharge database (HA), Outpatients Services (OPS), Drug Pharmacy database (DP) and Hospital Drug database (HD), by category, subcategory and phase of care. Epicost-2 prevalence population of 12,157 TC patients.

Database	Service	Category	Subcategory	Initial		Continuing		Final	
				N	%	N	%	N	%
**HA**	**Diagnoses**	Diagnosis and monitoring	Diagnosis	1599	82.2	6647	80.6	120	58.0
			Monitoring	125	6.4	864	10.5	82	39.6
		Radiotherapy	Radiotherapy	216	11.1	656	8.0	3	1.4
		Chemotherapy	Chemotherapy	6	0.3	82	1.0	2	1.0
		**Total**		**1946**	**100.0**	**8249**	**100.0**	**207**	**100.0**
	**Procedures**	Diagnosis and monitoring	High diagnostic	132	6.9	382	4.8	28	20.4
			Diagnosis	37	1.9	250	3.1	7	5.1
			Monitoring	5	0.3	69	0.9	4	2.9
		Surgery	Total Thyroidectomy	915	47.9	3530	44.0	19	13.9
			Lymphadenectomy	179	9.4	336	4.2	17	12.4
			Partial Thyroidectomy	124	6.5	404	5.0	3	2.2
			Other Surgery	30	1.6	107	1.3	6	4.4
			Monitoring	14	0.7	94	1.2	2	1.5
		Radiotherapy	Radiotherapy	417	21.8	2339	29.2	20	14.6
		Support therapy	Support therapy	58	3.0	504	6.3	31	22.6
		**Total**		**1911**	**100.0**	**8015**	**100.0**	**137**	**100.0**
**OPS**	**Procedures**	Diagnosis and monitoring	Blood test	60,864	81.2	641,168	85.0	5,968	82.9
			Diagnosis	3778	5.0	36,172	4.8	217	3.0
			Monitoring	3307	4.4	27,967	3.7	325	4.5
			Biopsy	2206	2.9	6505	0.9	67	0.9
			High diagnostics	1125	1.5	9145	1.2	248	3.4
			Specialist examination	1059	1.4	9110	1.2	117	1.6
		Radiotherapy	Radiotherapy	1623	2.2	16,630	2.2	211	2.9
		Support therapy	Support therapy	978	1.3	7232	1.0	48	0.7
		**Total**		**74,940**	**100.0**	**753,929**	**100.0**	**7,201**	**100.0**
**DP**	**Drugs**	Thyroid hormones		12,028	53.1	202,778	60.0	1744	52.7
		Support therapy		8376	37.0	122,887	36.3	1074	32.5
		Antibiotics		101	0.4	594	0.2	6	0.2
		Antithrombotics		1	0.0	31	0.0	0	0.0
**HP**	**Drugs**	Antithrombotics		907	4.0	5773	1.7	305	9.2
		Support therapy		827	3.7	4158	1.2	52	1.6
		Thyroid hormones		206	0.9	787	0.2	17	0.5
		Antibiotics		135	0.6	805	0.2	24	0.7
		Chemotherapy		42	0.2	249	0.1	85	2.6
		Analgesics		33	0.1	51	0.0	1	0.0
		**Total**		**22,656**	**100.0**	**338,113**	**100.0**	**3308**	**100.0**

**Figure 3.**
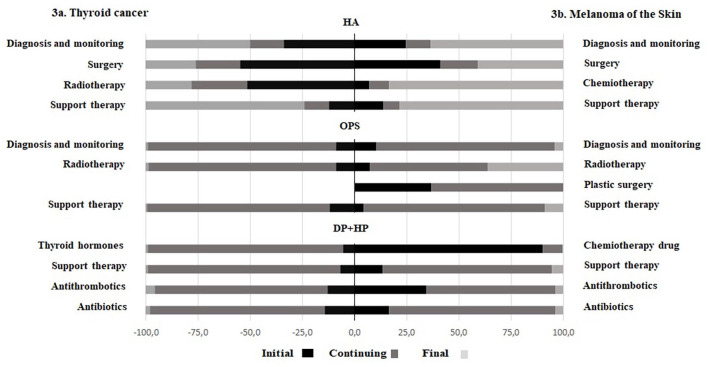
(a) Thyroid cancer; (b) Melanoma of the skin. Percent distribution of services provided to thyroid cancer (TC) and melanoma of the skin (MS) patients, four main categories by phase of care, in Hospital Admissions and Discharges (HA), Outpatients (OPS), Pharmacy Drugs (DP), Hospital Drugs (HD) databases. Epicost-2 prevalence population.

Figure 3a shows the percentage distribution of the four main categories in HSD for TC patients by phases of care. Each category is weighed by the percentage distribution of patients by phase of care. ‘Surgery’ (54.8%) and ‘Radiotherapy’ are mostly performed in hospitals (51.4%) in the initial phase and as outpatients’ services (90.1%) in the continuing phase. In outpatients, ‘Diagnosis and monitoring’ (90.2%), ‘Radiotherapy’ (90.2%) and ‘Support therapy’ (87.6%) procedures are most frequently performed in continuing phase. Pharmacy drug prescriptions are mostly used in continuing phase: ‘Thyroid hormones’ (93.6%), ‘Support therapy’ (92.5%), ‘Antibiotics’ (84.0%) and ‘Antithrombotics’ (82.7%).

### Health services provided to MS cases in the Epicost-2 population

The 14,924 prevalent cases of MS in the Epicost2 population, according to the phase of care, are: 1691 patients in the initial phase (11.3%), 12,910 in the continuing phase (86.5%) and 323 in the final one (2.2%), respectively. Table 3 shows the frequencies of hospitalizations, OPS services and drugs provided to MS prevalent cases, ordered by *i*-codes, in each phase of care. We grouped the fine-tuning diagnosis/procedures/drugs according to clinically relevant categories and/or subcategories by HSD. There are 18,833 hospital discharges collected in the HA database provided for 14,924 MS patients, by phase of care: 10,914 discharges have at least one cancer-related procedure while 7919 discharges have cancer-related diagnoses but no corresponding cancer-related procedure from 1 January 2014 to 31 December 2018. The procedures mainly administrated in ambulatory is the ‘Diagnosis and monitoring’ category, from 97.6% in the initial/continuing phase of care to 82.9% in the final one, with the three common ‘Specialist examination’, ‘High diagnostics’, ‘Biomarker’ subcategories. The prescriptions of drugs collected in the HP and DP databases for MS patients are: Antithrombotics (24.6%) and Chemotherapy (23.0%) for MS are mainly given in hospital pharmacies, in the initial phase. In the continuing and the final phase, the top three common categories of prescriptions for drugs are ‘Support therapy’, ‘Antithrombotics’ and ‘Analgesics’ collected in the DP database.

**Table 3.** Diagnoses and procedures provided for Skin Melanoma (MS) cancer patients, collected in the: Hospital Admission and Discharge Database (HA), Outpatients Services (OPS), Drug Prescriptions database (DP) and Hospital Drug database (HD), described by category, subcategory and phase of care. Epicost-2 prevalence population of 14,924 S patients.

Database	Service	Category	Subcategory	Initial		Continuing		Final	
				N	%	N	%	N	%
**HA**	**Diagnoses**	Diagnosis and monitoring	Diagnosis	1574	97.3	5142	91.4	591	87.6
		Chemotherapy		26	1.6	353	6.3	72	10.7
		Surgery	Surgery	14	0.9	88	1.6	12	1.8
			Plastic surgery	4	0.2	43	0.8	0	0.0
		**Total**		**1618**	**100.0**	**5626**	**100.0**	**675**	**100.0**
	**Procedures**	Diagnosis and monitoring	Cardiologic assessment	91	4.4	600	7.5	106	12.1
			High diagnostic	88	4.3	517	6.5	117	13.3
			Monitoring	50	2.4	271	3.4	28	3.2
			Conventional radiology	46	2.2	188	2.4	54	6.2
			Ultrasonography	40	2.0	199	2.5	29	3.3
			Other diagnostic procedure	23	1.1	108	1.4	5	0.6
			Biopsy	5	0.2	64	0.8	12	1.4
		Surgery	Surgery	858	41.9	3010	37.7	138	15.7
			Lymphadenectomy	415	20.3	1165	14.6	93	10.6
			Plastic Surgery	255	12.5	830	10.4	48	5.5
			Other surgery	9	0.4	36	0.5	3	0.3
		Chemotherapy	Chemotherapy	19	0.9	111	1.4	31	3.5
		Radiotherapy	Radiotherapy	5	0.2	36	0.5	11	1.3
		Biologic therapy	Biologic therapy	13	0.6	290	3.6	52	5.9
		Transfusion	Transfusion	23	1.1	109	1.4	35	4.0
		Support therapy	Support therapy	108	5.3	454	5.7	116	13.2
		**Total**		**2048**	**100.0**	**7988**	**100.0**	**878**	**100.0**
**OPS**	**Procedures**	Diagnosis and monitoring	Specialist examination	472	30.8	3443	27.3	149	21.4
			High diagnostics	240	15.7	1699	13.5	125	18.0
			Bio-marker	205	13.4	2084	16.6	50	7.2
			Genetic marker	138	9.0	1496	11.9	37	5.3
			Culture test	114	7.4	1141	9.1	58	8.3
			Physiatry	103	6.7	674	5.4	9	1.3
			Ultrasonography	83	5.4	481	3.8	11	1.6
			Blood test	68	4.4	876	7.0	96	13.8
			Biopsy	46	3.0	228	1.8	26	3.7
			Genetic test	26	1.7	163	1.3	14	2.0
			Endoscopic Procedure	1	0.1	5	0.0	1	0.1
		Plastic surgery		7	0.5	12	0.1	0	0.0
		Surgery		3	0.2	38	0.3	0	0.0
		Radiotherapy		23	1.5	172	1.4	111	16.0
		Support therapy		4	0.3	79	0.6	8	1.2
		**Total**		**1533**	**100.0**	**12,591**	**100.0**	**695**	**100.0**
**DP**	**Drugs**	Support therapy		7322	12.9	54,274	33.8	3613	33.4
		Antithrombotics		4645	8.2	32,173	20.0	1988	18.4
		Antibiotics		3244	5.7	21,758	13.6	1000	9.3
		Analgesics		3205	5.6	24,193	15.1	1939	18.0
		Cortisone		1632	2.9	10,369	6.5	1430	13.2
		Glaucoma medication		1036	1.8	4843	3.0	236	2.2
		Antidepressant		642	1.1	5041	3.1	267	2.5
		Chemotherapic drug		454	0.8	860	0.5	35	0.3
		Antihistamine		392	0.7	2592	1.6	68	0.6
		Antiasthmatic		114	0.2	1062	0.7	21	0.2
**HP**	**Drugs**	Antithrombotics		14,015	24.6	1419	0.9	136	1.3
		Chemotherapic drug		13,072	23.0	542	0.3	26	0.2
		Biologic therapy		3171	5.6	757	0.5	32	0.3
		Support therapy		1572	2.8	218	0.1	1	0.0
		Antibiotics		1269	2.2	120	0.1	7	0.1
		Cortisone		723	1.3	169	0.1	1	0.0
		Analgesics		418	0.7	78	0.0	2	0.0
		**Total**		**56,926**	**100.0**	**160,468**	**100.0**	**10,802**	**100.0**

Distribution of four main categories in HS database for MS - weighted by the percentage distribution of patients by phase of care - are shown in Figure 3b. In hospital setting, ‘Surgery’ is carried out in hospital mainly in the initial phase of care (41.2%) and in final (40.8%); ‘Chemotherapy’ in the final (83.6%). In OPS, ‘Radiotherapy’ is mostly performed in the continuing (56.2%) and the final (36.3%) phase of care, ‘Diagnosis and monitoring’ procedures are most frequently performed in continuing phase (85.6%), while ‘Plastic surgery’ in the initial (36.8%) and the continuing (63.2%). ‘Support therapy’ is mostly administered in the final phase in hospitals (78.4%), in the continuing in OPS (86.8%), in hospital/territorial pharmacies (81.3%). Percent distribution of other hospital/territorial pharmacy drug prescriptions vary among the phase of care. The highest percentages are: 90.2% for ‘Chemotherapic drug’ in the initial phase, 79.9% for ‘Antibiotics’ and 61.8% for ‘Antithrombotics’ in the continuing one.

### The 10 top cancer-related health services for TC patients in the Epicost-2 prevalence population

Table 1B in Online Appendix B shows the top 10 procedures provided to TC patients during hospitalizations, with description, category and subcategory by phase of care. ‘Surgery’ and ‘Radiotherapy’ are the most common categories, accounting for 51.5% and 19.6% respectively, in the initial phase. In the continuing phase of care ‘Surgery’ and ‘Radiotherapy’ are the most common categories with 42.8% and 20.8% respectively. The subcategory ’Total thyroidectomy’ among the ‘Surgery’ procedures, takes the first rank with 37.1% and 29.9% respectively in the initial and continuing phase. In the final phase, ‘Diagnosis and monitoring’ represents the most common category, with the subcategory ‘Hight diagnostic’, accounting for 24.0%. The second most common category is ‘Surgery’ with 21.4%, followed by ‘Support therapy’ with 20.9% and ‘Radiotherapy’ with 11.2%. In Online Appendix Table 2B there the top 10 procedures are provided in the ambulatories to TC patients. Note that the ‘Blood test’ subcategory with the typical routine components of TC monitoring (i.e. Venous blood sampling, Thyrotropin, Thyroxine, Triiodothyronine total, Calcium, Tyroglobulin, Anti Thyroglobulin antibody, Vitamin D, Calcium) according to the clinical guidelines, is present in the three phases accounting for 72.5%, 77.1%, 72.7% respectively followed by ‘Ultrasound diagnostic’ of the head and neck procedure in the initial (3.8%) and continuing (4.4%) phase. Online Appendix Table 3B shows the top 10 drug prescriptions collected in HD and DP databases. Grouping on category, ‘Thyroid Hormones’ is the most common in all phases while ‘Support Therapy’ is second. In particular, Levothyroxine sodium and Liothyronine sodium account for 51.9% in the initial phase, 60.2% in the continuing, and 53.2% in the final phase, while supplements that prevents low levels of calcium or/and vitamin D (Colecalcifediol; Calcifediol; Calcitriol; Calcium, combinations with vitamin D and/or other drugs; Calcium (different salts in combination);Calcium carbonate) account for 39.6% in initial phase, 37.4% in continuing, 33.3% in final one.

### The top 10 cancer-related health services for MS patients in the Epicost-2 prevalence population

The top 10 procedures collected in HA provided to MS patients, are shown in Table 4B in Online Appendix B, by phase of care. In the initial and continuing phase of care ‘Surgery’ is the most common category in the hospitalizations, with 52.5%, 41.4% respectively. ‘Diagnosis and monitoring’ is the second most common procedure ranging from 9.2%, 12.5%, for initial and continuing phase of care respectively. In the final phase of care, ‘Diagnosis and monitoring’ (mainly ‘Conventional radiology’ and ‘Cardiologic assessment’ subcategories) ranks first (26.2%), followed by ‘Surgery’ (12.2%) and ‘Support therapy’ (11.6%) with ‘Injection or infusion of other therapeutic or prophylactic substances’ and ‘Injection of antibiotics’. In Online Appendix Table 5B are the top 10 procedures provided to MS patients collected in the OPS database. The ‘Diagnosis and monitoring’ category with the typical routine components of MS monitoring (‘Neurological visit’, ‘Biomarker’, ‘Genetic marker’, ‘Culture test’ subcategories) is present in the three phases accounting for 62.1%, 65.7%, 56.3% respectively. Online Appendix Table 6B shows the top 10 prescriptions for drugs collected in HD and DP databases for MS patients. In the initial phase, grouping the categories, the top three most frequent category of drugs are ‘Support therapy’ (23.0%) mainly gastroprotector, ‘Antithrombotics’ (19.9%) and ‘Chemotherapic drugs’ (12.5%) -Interferon alfa-2a and Interferon alfa-2b. In the final phase, are ‘Antithrombotics’ (57.2%) and ‘Biologic therapy’ (46.5%) and ‘Chemotherapy drug’ (22.2%)- Temozolomide, Interferon alfa-2a.

## Discussion

In this paper, we propose an indirect approach for the identification of the services provided by the INHS for cancer care, along the phases of care. This indirect approach is typically used in studies of cancer costs in the United States, based on the linkage of SEER (Surveillance, Epidemiology, and End Result Program) and Medicare databases^16^ where a non-cancer sample in Medicare (controls) was used to identify payments for service related to cancer diagnosed cancer (cases), collected in SEER. Comparison between these groups of patients allows us to isolate the incremental cost due to cancer.^27,28^

As a comparison cohort is not directly available for CRs, we developed a study design borrowed from nested case-control and SCC observational studies^17-19^ and adapted to our data to apply an indirect approach. This study design is particularly appropriate in our case, since data from electronic health records do not collect information about possible confounding factors which might limit the matching between cases and controls. The method has been already applied for validation purposes to the case of breast cancer within the Epicost study, first edition, finding over 97% of concordance.^15^ Moreover, a validation of the statistically significant lists of *i*-codes by the panel of expert oncologists and clinicians guarantees that final lists are consistent with the clinical practice and current guidelines. We found the following results: for TC patients in the initial phase of care, ‘Surgery’ is the first cause of hospitalization, followed by ‘Radiotherapy’ and ‘Diagnosis and monitoring’. The ‘Support therapy’ (Calcium and/or Vitamin D) is either prescribed in the final phase in hospital or in the continuing phase in ambulatory. In the continuing phase, the ambulatory services ‘Diagnosis and monitoring’, ‘Radiotherapy’ are equally distributed and are due to possible diseases progression. The drugs prescribed to patients are mainly common in the continuing phase of care and are ‘Thyroid hormones’, ‘Antithrombotics’ and ‘Antibiotics’. For MS patients, in the initial and in final phase probably due to diseases progression, ‘Surgery’ and ‘Diagnosis and monitoring’ are the main cause of hospitalization. In the final phase, the therapies mainly administered in hospital are ‘Chemotherapy’ (Injection or infusion of chemotherapeutic substance) and ‘Support therapy’ (Injection or infusion of other therapeutic or prophylactic substances and Injection of antibiotics). In the continuing phase, the categories of ambulatory procedure are mainly ‘Diagnosis and monitoring’, ‘Support therapy’, followed by ‘Plastic surgery’ and ‘Radiotherapy’. In the initial phase ‘Chemotherapic drug’ (Interferon alfa-2a and Interferon alfa-2b) is the main common prescription, while in the continuing phase it is ‘Support therapy’, ‘Antithrombotics’ and ‘Antibiotics’.

We believe that the steps described below allow this study to be replicated on other cancer-sites and related health services provided, in Italy, and in other areas covered by registration:

- to calculate a prevalent population of cases collected by Italian cancer registries (CRs) at certain date X and followed-up after until date X+1year;
- to design a self-controlled crossover on cancer cases (cancer Y) drawn from the above prevalent population of cases, with diagnosis in ((X-6 months)date-Xdate), followed-up in the 12 months period after the diagnosis; controls (1:1) are paired patients, observed in the 12-month period from 18 months to six months before cancer diagnosis.
- to link the prevalent cases of controlled crossover study to available claims for HS collected in a period of time compatible and transmitted to the Italian regional health authority to be reimbursed;
- to compare the frequency of health services (HS) provided over time to patients it is possible identify the potentially cancer-related services that showed a statistically significant increase (p<=5%) after diagnosis;
- to place the contribution of oncology and clinical experts, firstly based on clinical and then on mathematical/economic criteria, after the statistical analysis to have validated lists of cancer-related services provided to patients affected by cancer Y.

A limitation of this study is related to the fact that these lists of cancer related procedures and drugs are obtained from HS database and refer to a period spanning from 1 January 2014 to 31 December 2018. Nowadays there has been a great improvement in drugs for TC: e.g. tyrosine inhibitors, such as sorafenib, sunitinib, lenvatinib, pazopanib, vandetanib, salpercatinib and cabozantinib; angiogenesis inhibitors, which act by counteracting the formation of new blood vessels that would be used for the nutrition of malignant cells. Some tyrosine kinase inhibitors - such as sunitinib, sorafenib, etc. - are also able to exert this action.^29,30^Likewise for the treatment of MS. In fact, new expensive classes of drugs have been used in recent years such as small-molecule inhibitors (BRAF and/or MEK) and immune checkpoint inhibitors (anti-PD1 and/or anti-CTLA4 monoclonal antibodies), that appear to improve progression-free survival of patients with metastatic.^31-33^ These new drugs are not included in the resulting lists (Online Appendix A). Moreover, the HS database includes procedure and drugs administered in the public health system. However, in Italy, the private sector is complementary to the public sector, so procedures/treatments performed exclusively in the private sectors (without any reimbursement by the NIHS) and not included in Epicost-2 project could be considered negligible.

The strengths of the present work are represented using individual data from administrative sources of health care services and from surveillance data in areas covered by population-based CRs, both based on individual data. This was possible thanks to the collaboration of the Italian Regions and ICRs involved, and the collective effort of experts in tumor registration, experts in oncology, in statistic, economics, and pharmacoepidemiology. In the Epicost-2 project, the availability of the HSD related to prevalent cases collecting in ICRs data from four years before to one year after prevalence date allowed us to apply the indirect method to overcome the limitations of the previous experience. The regional HSDs are based on well-established data collection procedures and classification systems that are used consistently across regions to ensure completeness and geographical comparability of data.^34^

In summary, we were able to identify health services along the path of prevalent TC and MS prevalent cancer cases in Italy, with the following advantages of:

- conduct of a study that concerns the entire prevalent cancer population, an essential element when the clinical studies available are few and mainly on a subgroup of patients^6,7^;
- choosing a study design, based on intra-person comparison, which allows control for known and unknown confounders that do not vary over time;
- use a statistical procedure that ensures error detection in the *Δ_i_* estimates (p<=0.05) from a statistical point of view;
- reducing the workload of clinicians and oncologists, who are requested to validate *i*-codes related to a specific cancer.

By comparing lists of cancer-related services obtained in both the Epicost and Epicost-2, we can estimate that using the indirect approach, the time requested of the panel of experts in respect to the fine-tuning of the lists is about 1/4 of that when using the direct approach.

A further improvement of the methodology could be to apply i-code lists based on patients’ clinical variables (a comorbidity indicator, age, sex and stage at diagnosis) which could influence choices between available treatments and drugs.

## Conclusions

The obtained lists of cancer-related diagnosis, procedures and treatments might be applicable to other areas or countries where population-based cancer registry data are available, if health services care data are available, classified according to the same international classification systems and/or conversion tables are available and can be linked at the individual level. The application of these lists has the advantage of producing consistent results thus enhancing the comparability of cost estimates.

## Supplemental Material

sj-pdf-1-tmj-10.1177_03008916251353109 – Supplemental material for An indirect approach to identify the healthcare services for thyroid and melanoma cancer patients in Italy: Epicost-2 project Supplemental material, sj-pdf-1-tmj-10.1177_03008916251353109 for An indirect approach to identify the healthcare services for thyroid and melanoma cancer patients in Italy: Epicost-2 project by Sandra Mallone, Andrea Tavilla, Tania Lopez, Daniela Pierannunzio, Luigino Dal Maso, Stefano Guzzinati, Ugo Fedeli, Alberto Gagliani, Alessandra Buja, Manuel Zorzi, Mario Fusco, Federica Toffolutti and Silvia Francisci in Tumori Journal

sj-pdf-2-tmj-10.1177_03008916251353109 – Supplemental material for An indirect approach to identify the healthcare services for thyroid and melanoma cancer patients in Italy: Epicost-2 project Supplemental material, sj-pdf-2-tmj-10.1177_03008916251353109 for An indirect approach to identify the healthcare services for thyroid and melanoma cancer patients in Italy: Epicost-2 project by Sandra Mallone, Andrea Tavilla, Tania Lopez, Daniela Pierannunzio, Luigino Dal Maso, Stefano Guzzinati, Ugo Fedeli, Alberto Gagliani, Alessandra Buja, Manuel Zorzi, Mario Fusco, Federica Toffolutti and Silvia Francisci in Tumori Journal

## References

[1] Cancer Today. Estimated number of prevalent cases, Both sexes, in 2022, https://gco.iarc.fr/today/en/dataviz/bars-prevalence?mode=cancer&key=total&cancers=16&sort_by=value1&prev_time=5&group_populations=1 (accessed 19 June 2025).

[2] De Angelis R Demuru E Baili P , et al. Complete cancer prevalence in Europe in 2020 by disease duration and country (EUROCARE-6): a population-based study. Lancet Oncol 2024; 25: 293–307. 38307102 10.1016/S1470-2045(23)00646-0

[3] Guzzinati S Toffolutti F Francisci S , et al. Patients with cancer who will be cured and projections of complete prevalence in Italy from 2018 to 2030. ESMO Open 2024; 9: 103635. 39043021 10.1016/j.esmoop.2024.103635 PMC11321301

[4] Johnston K Levy AR Lorigan P , et al. Economic impact of healthcare resource utilisation patterns among patients diagnosed with advanced melanoma in the United Kingdom, Italy, and France: Results from a retrospective, longitudinal survey (MELODY study). Eur J Cancer 2012; 48: 2175–2182. 22480965 10.1016/j.ejca.2012.03.003

[5] Li M Meheus F Polazzi S , et al. The economic cost of thyroid cancer in France and the corresponding share associated with treatment of overdiagnosed cases. Val Health 2023; 26: 1175–1182. 10.1016/j.jval.2023.02.016 36921898

[6] Buja A Rivera M De Polo A , et al. Differences in direct costs of patients with stage I cutaneous melanoma: A real-world data analysis. Eur J Surg Oncol 2020; 46: 976-981. 32146052 10.1016/j.ejso.2020.02.017

[7] Buja A Cozzolino C Zanovello A , et al. Cost items in melanoma patients by clinical characteristics and time from diagnosis. Front Oncol 2023; 13: 1-13. 10.3389/fonc.2023.1234931 PMC10666743 38023154

[8] Brown ML Riley GF Potosky AL , et al. Obtaining long-term disease specific costs of care: application to Medicare enrollees diagnosed with colorectal cancer. Med Care 1999; 37: 1249-1259. 10599606 10.1097/00005650-199912000-00008

[bibr9-03008916251353109] Yabroff KR Lamont EB Mariotto A , et al. Cost of care for elderly cancer patients in the United States. JNCI 2008; 100: 630–641. 18445825 10.1093/jnci/djn103

[bibr10-03008916251353109] De Oliveira C Weir S Rangrej J , et al. The economic burden of cancer care in Canada: a population-based cost study. CMAJ Open 2018; 6: E1–10. 10.9778/cmajo.20170144 PMC5878959 29301745

[11] Laudicella M Walsh B Burns E , et al. Cost of care for cancer patients in England: evidence from population-based patient-level data. Brit J Cancer 2016; 114: 1286–1292. 27070711 10.1038/bjc.2016.77 PMC4891510

[12] Francisci S Guzzinati S Mezzetti M , et al. Cost profiles of colorectal cancer patients in Italy based on individual patterns of care. BMC Cancer 2013; 13: 329. 23826976 10.1186/1471-2407-13-329 PMC3706387

[13] Lopes JM Rocha-Gonçalves F Borges M , et al. The cost of cancer treatment in Portugal. Ecancermedicalsci 2017; 11: 765. 10.3332/ecancer.2017.765 PMC5606294 28955401

[14] Massa I Balzi W Burattini C , et al. The challenge of sustainability in healthcare systems: Frequency and cost of inappropriate patterns of breast cancer care (the E.Pic.A study). Breast 2017; 34: 103–107. 28558338 10.1016/j.breast.2017.05.007

[15] Busco S Tavilla A Gigli A , et al. A direct method for the identification of patterns of care using administrative databases: the case of breast cancer. Eur J Health Economic 2021; 22: 1477–1485. 10.1007/s10198-021-01327-8 PMC8558165 34312745

[16] Mariotto AB Enewold L Zhao J , et al. Medical care costs associated with cancer survivorship in the United States. Cancer Epidemiol Biomark Prevent 2020; 29: 1304-1312. 10.1158/1055-9965.EPI-19-1534 PMC9514601 32522832

[17] Ernster VL. Nested case-control studies. Prevent Med 1994; 23: 587–590. 10.1006/pmed.1994.1093 7845919

[bibr18-03008916251353109] Iwagami M Takeuchi Y. Introduction to self-controlled study design. Ann Clin Epidemiol 2021; 3: 67–73.

[19] Cadarette SM Maclure M Delaney JAC , et al. Control yourself: ISPE-endorsed guidance in the application of self-controlled study designs in pharmacoepidemiology. Pharmacoepidemiol Drug Safety 2021; 30: 671-684. 10.1002/pds.5227 PMC8251635 33715267

[20] World Health Organization. International Classification of Disease, 10th revision. ICD10-CM. Ginevra: WHO, 2007.

[21] World Health Organization. International Classification of Diseases, 9th revision. ICD9-CM. Ginevra: WHO, 2007.

[22] World Health Organization. Collaborating Centre for Drug Statistics Methodology. Anatomical Therapeutic Chemical (ATC) classification system, ATC/DDD, http://www.whocc.no/ (Last updated: 2025-06-10)

[23] Francisci S Capodaglio G Gigli A , et al. Cancer cost profiles: The Epicost estimation approach. Front Pub Health 2022; 10: 1-13. 10.3389/fpubh.2022.974505 PMC9533128 36211660

[24] Gigli A Francisci S Capodaglio G , et al. The economic impact of rectal cancer: a population-based study in Italy. Int J Environ Res Pub Health 2021; 18: 474. 33430156 10.3390/ijerph18020474 PMC7827442

[25] Decreto del Presidente del Consiglio dei Ministri, March 3, 2017, Identificazione dei sistemi di sorveglianza e dei registri di mortalità, di tumori e di altre patologie, 17A03142, GU Serie Generale n.109 del 12-05-2017, www.gazzettauffiiale.it/eli/id/2017/05/12/17A03142/sg, (2017, accessed 26 November 2021).

[26] SAS. Copyright (c) 2002-2012. Cary, NC, USA: SAS Institute Inc, 2012.

[27] Akobundu E Ju J Blatt L , et al. Cost-of-Illness studies. PharmacoEconomics 2006; 24: 869–890. 16942122 10.2165/00019053-200624090-00005

[28] Caulley L Thavorn K Rudmik L , et al. Direct costs of adult chronic rhinosinusitis by using 4 methods of estimation: Results of the US Medical Expenditure Panel Survey. J Allergy Clin Immunol 2015; 136: 1517–1522. 26483176 10.1016/j.jaci.2015.08.037

[29] Fallahi P Ferrari SM Galdiero MR , et al. Molecular targets of tyrosine kinase inhibitors in thyroid cancer. Semin Cancer Biol 2022; 79: 180-196. 33249201 10.1016/j.semcancer.2020.11.013

[30] Gild ML Tsang VHM Clifton-Bligh RJ , et al. Multikinase inhibitors in thyroid cancer: timing of targeted therapy. Nat Rev Endocrinol 2021; 17: 225–234. 33603220 10.1038/s41574-020-00465-y

[31] Pasquali S Hadjinicolaou AV Chiarion Sileni V , et al. Systemic treatments for metastatic cutaneous melanoma (Review). Cochrane Database Systematic Rev 2018; 2: CD011123. 10.1002/14651858.CD011123.pub2 PMC6491081 29405038

[32] Berk-Krauss J Stein JA Weber J , et al. New systematic therapies and trends in cutaneous melanoma deaths among US whites, 1986-2016. Am J Public Health 2020; 110: 731-733. 32191523 10.2105/AJPH.2020.305567 PMC7144422

[33] Mason R Au L Ingles Garces A , et al. Current and emerging systemic therapies for cutaneous metastatic melanoma. Expert Opin Pharmacother 2019; 20: 1135-1152. 31025594 10.1080/14656566.2019.1601700

[34] Pierannunzio D Fedeli U Francisci S , et al. Thyroidectomies in Italy: A population-based national analysis from 2001 to 2018. Thyroid 2022; 32: 263-272. 35018816 10.1089/thy.2021.0531

