# Supplementary material for: An indirect approach to identify the healthcare services for thyroid and melanoma cancer patients in Italy: Epicost-2 project
Source: Tumori. 2025 Sep 4;111(5):400–12. doi: 10.1177/03008916251353109 (PMC12476483; doi:10.1177/03008916251353109)
Supplement: sj-pdf-1-tmj-10.1177_03008916251353109 – Supplemental material for An indirect approach to identify the healthcare services for thyroid and melanoma cancer patients in Italy: Epicost-2 project [file sj-pdf-1-tmj-10.1177_03008916251353109.pdf]

Appendix A Thyroid cancer - clinically valitaded list of services in HSD sorted by code

Table 1A Thyroid cancer-related diagnoses (58) by ICD9-CM, description, category, subcategory in Hospital Admissions and Discharges database (HA). Self-controlled crossover study in Epicost-2 project: 3,017 prevalent cases (diagnosed in 1/07/2015-31/12/2017; followed-up to 31/12/2018) and their 5,534 paired records collected in the HA database.

DIAGNOSES

ICD9-CM CODE	DESCRIPTION	CATEGORY	SUBCATEGORY
193	Malignant noeplasm of thyroid gland	Diagnosis and monitoring	Diagnosis
195.0	Malignant neoplasm of head, face and neck	Diagnosis and monitoring	Diagnosis
196.0	Secondary and unspecified malignant neoplasm of lymph nodes of head face and neck	Diagnosis and monitoring	Diagnosis
196.1	Secondary and unspecified malignant neoplasm of intrathoracic lymph nodes	Diagnosis and monitoring	Diagnosis
196.3	Secondary and unspecified malignant neoplasm of axilla and upper limb lymph nodes	Diagnosis and monitoring	Diagnosis
196.8	Secondary and unspecified malignant neoplasm of lymph nodes of multiple regions	Diagnosis and monitoring	Diagnosis
196.9	Secondary and unspecified malignant neoplasm of lymph node, unspecified.	Diagnosis and monitoring	Diagnosis
197.0	Secondary malignant neoplasm of lung	Diagnosis and monitoring	Diagnosis
198.5	Secondary malignant neoplasm of bone	Diagnosis and monitoring	Diagnosis
226	Benign neoplasm of thyroid glands	Diagnosis and monitoring	Diagnosis
227.1	Benign neoplasm of parathyroid gland	Diagnosis and monitoring	Diagnosis
237.4	Neoplasm of uncertain behavior of other and unspecified endocrine glands	Diagnosis and monitoring	Diagnosis
239.7	Neoplasm of unspecified nature of endocrine glands and other parts of nervous system	Diagnosis and monitoring	Diagnosis
240.0	Goiter specified as simple	Diagnosis and monitoring	Diagnosis
240.9	Goiter unspecified	Diagnosis and monitoring	Diagnosis
241.0	Nontoxic nodular goiter	Diagnosis and monitoring	Diagnosis

241.1	Nontoxic multinodular goiter	Diagnosis and monitoring	Diagnosis
241.9	Unspecified nontoxic nodular goiter	Diagnosis and monitoring	Diagnosis
242.00	Toxic diffuse goiter	Diagnosis and monitoring	Diagnosis
242.10	Thyrotoxicosis with toxic single thyroid nodule without thyrotoxic crisis or storm.	Diagnosis and monitoring	Diagnosis
242.20	Toxic multinodular goiter without thyrotoxic crisis or storm	Diagnosis and monitoring	Diagnosis
244.0	Acquired hypothyroidism	Diagnosis and monitoring	Monitoring
244.3	Other iatrogenic hypothyroidism	Diagnosis and monitoring	Monitoring
244.8	Other specified acquired hypothyroidism	Diagnosis and monitoring	Monitoring
244.9	Neoplasm of uncertain behavior of other and unspecified endocrine glands	Diagnosis and monitoring	Diagnosis
245.1	Subacute thyroiditis	Diagnosis and monitoring	Diagnosis
245.2	Chronic lymphocytic thyroiditis	Diagnosis and monitoring	Diagnosis
245.3	Chronic fibrous thyroiditis	Diagnosis and monitoring	Diagnosis
245.8	Other and unspecified chronic thyroiditis	Diagnosis and monitoring	Diagnosis
245.9	Thyroiditis unspecified	Diagnosis and monitoring	Diagnosis
246.2	Cyst of thyroid	Diagnosis and monitoring	Diagnosis
246.8	Other specified disorders of thyroid	Diagnosis and monitoring	Diagnosis
246.9	Disorder of thyroid, unspecified.	Diagnosis and monitoring	Diagnosis
252.00	Hyperparathyroidism, unspecified	Diagnosis and monitoring	Diagnosis
252.01	Primary hyperparathyroid	Diagnosis and monitoring	Diagnosis
252.1	Hypoparathyroidism	Diagnosis and monitoring	Monitoring
275.41	Hypocalcemia	Diagnosis and monitoring	Monitoring
338.3	Neoplasm related pain	Diagnosis and monitoring	Monitoring
376.21	Thyrotoxic exophthalmos	Diagnosis and monitoring	Diagnosis
401.1	Essential (primary) hypertension	Diagnosis and monitoring	Monitoring
401.9	Unspecified essential hypertension	Diagnosis and monitoring	Monitoring
427.31	Atrial fibrillation	Diagnosis and monitoring	Monitoring
478.30	Paralysis of vocal cords or larynx, unspecified	Diagnosis and monitoring	Monitoring
478.31	Paralysis of vocal cords and larynx, unilateral	Diagnosis and monitoring	Monitoring
478.32	Unilateral paralysis of vocal cords or larynx, complete	Diagnosis and monitoring	Monitoring

478.33	Paralysis of vocal cords and larynx, bilateral	Diagnosis and monitoring	Monitoring
478.6	Edema of larynx	Diagnosis and monitoring	Monitoring
785.6	Enlargement of lymph nodes	Diagnosis and monitoring	Diagnosis
998.11	Hemorrhage complicating a procedure	Diagnosis and monitoring	Monitoring
998.12	Hematoma complicating a procedure	Diagnosis and monitoring	Monitoring
998.59	Other postoperative infection	Diagnosis and monitoring	Monitoring
V10.87	Personal history of malignant neoplasm of thyroid.	Diagnosis and monitoring	Diagnosis
V10.88	Personal history of malignant neoplasm of other endocrine glands and related structures	Diagnosis and monitoring	Diagnosis
V12.2	Personal history of endocrine metabolic and immunity disorders	Diagnosis and monitoring	Diagnosis
V45.89	Other postsurgical status	Diagnosis and monitoring	Monitoring
V58.0	Encounter for radiotherapy	Radiotherapy	Radiotherapy
V58.11	Encounter for antineoplastic chemotherapy	Chemotherapy	Chemotherapy
V58.42	Aftercare following surgery for neoplasm	Chemotherapy	Chemotherapy

Table 2A Thyroid cancer-related procedures (50) by ICD9-CM, description, category, subcategory in Hospital Admissions and Discharges database (HA).
Self-controlled crossover study in Epicost-2 project: 3,017 prevalent cases (diagnosed in 1/07/2015-31/12/2017; followed-up to 31/12/2018) and their 5,534 paired records collected in the HA database.

PROCEDURES

ICD9-CM CODE	DESCRIPTION	CATEGORY	SUBCATEGORY
06.01	Aspiration of thyroid field.	Surgery	Other Surgery
06.02	Reopen thyroid field wnd	Surgery	Monitoring
06.09	Other incision of thyroid field.	Surgery	Other Surgery
06.11	Closed (percutaneous) (needle) biopsy of thyroid gland	Diagnosis and monitoring	Diagnosis
06.12	Open biopsy of thyroid gland	Diagnosis and monitoring	Diagnosis
06.2	Unilateral Thyroid Lobectomy	Surgery	Partial Thyroidectomy
06.31	Excision of lesion of thyroid	Surgery	Partial Thyroidectomy
06.39	Other partial thyroidectomy	Surgery	Partial Thyroidectomy
06.4	Complete thyroidectomy	Surgery	Total Thyroidectomy
06.51	Partial substernal thyroidectomy	Surgery	Partial Thyroidectomy
06.52	Complete substernal thyroidectomy	Surgery	Total Thyroidectomy
06.6	Lingual thyroid excision	Surgery	Other Surgery
06.7	Excision of thyroglossal duct or tract. Thyrogloss duct excision.	Surgery	Other Surgery
06.81	Total parathyroidectomy	Surgery	Other Surgery
06.89	Other parathyroidectomy	Surgery	Other Surgery
06.91	Thyroid isthmus division	Surgery	Other Surgery
06.92	Ligation of thyroid vessels	Surgery	Other Surgery
06.95	Parathyroid tissue reimplantation	Surgery	Other Surgery

30.09	Other Excision Or Destruction Of Lesion Or Tissue Of Larynx	Surgery	Other Surgery
31.1	Temporary tracheostomy	Surgery	Other Surgery
31.42	Larygnoscopy/tracheoscop (Laryngoscopy and other tracheoscopy)	Diagnosis and monitoring	Diagnosis
31.48	Other diagnostic procedures on larynx	Diagnosis and monitoring	Diagnosis
31.5	Local excision or destruction of lesion or tissue of trachea	Surgery	Other Surgery
39.98	Control of hemorrhage, not otherwise specified.	Surgery	Monitoring
40.11	Biopsy of lymphatic structure	Diagnosis and monitoring	Diagnosis
40.21	Excision Of Deep Cervical Lymph Node	Surgery	Lymphadenectomy
40.3	Regional lymph node excision	Surgery	Lymphadenectomy
40.40	Radical neck dissection, not otherwise specified	Surgery	Lymphadenectomy
40.41	Radical neck dissection, unilateral	Surgery	Lymphadenectomy
40.42	Radical neck dissection, bilateral	Surgery	Lymphadenectomy
40.50	Radical excision of lymph nodes, not otherwise specified	Surgery	Lymphadenectomy
40.59	Radical excision of other lymph nodes	Surgery	Lymphadenectomy
86.4	Radical excision of skin lesion	Surgery	Monitoring
86.59	Suture Of Skin And Subcutaneous Tissue Of Other Sites.	Surgery	Other Surgery
87.03	Computerized axial tomography of head	Diagnosis and monitoring	High diagnostic
87.41	Computerized axial tomography of thorax	Diagnosis and monitoring	High diagnostic
88.71	Diagnostic ultrasound of head and neck	Diagnosis and monitoring	Diagnosis
88.97	Magnetic resonance imaging of other and unspecified sites)	Diagnosis and monitoring	High diagnostic
89.61	Systemic arterial pressure monitoring	Diagnosis and monitoring	Monitoring
90.11	Microscopic examination of specimen from endocrine gland, not elsewhere classified, bacterial smear.	Diagnosis and monitoring	Diagnosis
90.19	Microscopic examination of specimen from endocrine gland, not elsewhere classified, other microscopic examination.	Diagnosis and monitoring	Diagnosis
90.79	Microscopic examination of specimen from lymph node and of lymph, other microscopic examination	Diagnosis and monitoring	Diagnosis
92.01	Thyroid scan and radioisotope function studies	Diagnosis and monitoring	High diagnostic
92.14	Bone scan	Diagnosis and monitoring	High diagnostic
92.18	Total body scan	Diagnosis and monitoring	High diagnostic

92.28	Injection or instillation of radioisotopes	Radiotherapy	Radiotherapy
92.29	Other radiotherapeutic procedur	Radiotherapy	Radiotherapy
93.57	Application Of Other Wound Dressing	Diagnosis and monitoring	Monitoring
99.23	Injection Of Steroid	Support therapy	Support therapy
99.29	Injection Or Infusion Of Other Therapeutic Or Prophylactic Substance	Support therapy	Support therapy

Table 3A Thyroid cancer-related procedures (65) by ICD9-CM, description, category, subcategory in Outpatients Services database (OPS). Self-controlled crossover study in Epicost-2 project: 3,017 prevalent cases (diagnosed in 1/07/2015-31/12/2017; followed-up to 31/12/2018) and their 255,041 paired records collected in the OPS database.

PROCEDURES

ICD9-CM CODE	DESCRIPTION	CATEGORY	SUBCATEGORY
05.31	Injection of anesthecic into the sympathetic nerves for analgesia	Support therapy	Support therapy
06.01	Aspiration of thyroid field	Diagnosis and monitoring	Biopsy
31.42	Laryngoscopy and other tracheoscopy	Diagnosis and monitoring	Diagnosis
40.11	Biopsy of lymphatic structure	Diagnosis and monitoring	Biopsy
87.41	Computerized axial tomography of thorax	Diagnosis and monitoring	High diagnostics
89.7	General physical examination	Diagnosis and monitoring	Specialist examination
99.29	Injection or infusion of other therapeutic or prophylactic substance	Support therapy	Support therapy
06.11.1	[Percutaneus] Bipsy [Needle Biopsy] of the thyroid	Diagnosis and monitoring	Biopsy
06.11.2	Ultrasound-guided thyroid biopsy	Diagnosis and monitoring	Biopsy
40.19.1	Ultrasound-guided fine needle biopsy of lymph node	Diagnosis and monitoring	Biopsy
87.03.8	Computed tomography (CT) of neck, without and with contrast	Diagnosis and monitoring	High diagnostics
87.41.1	Computed tomography (CT) of thorax, without and with contrast	Diagnosis and monitoring	High diagnostics
88.01.6	Computed tomography (CT) of whole abdomen	Diagnosis and monitoring	High diagnostics
88.38.9	Total body CT for oncological staging without and with contrast medium	Diagnosis and monitoring	High diagnostics
88.71.4	Ultrasound diagnostics of the head and neck	Diagnosis and monitoring	Diagnosis
88.9.52	Magnetic resonance imaging (MRI) of brain and brain stem, without and with contrast	Diagnosis and monitoring	High diagnostics
88.90.2	Three Dimensional computed tomography (CT) reconstruction	Diagnosis and monitoring	High diagnostics

88.91.6	Nuclear magnetic resonance (MRI) of the neck	Diagnosis and monitoring	High diagnostics
88.91.7	Nuclear magnetic resonance (MRI) of the neck, without and with contrast	Diagnosis and monitoring	High diagnostics
88.94.P	MRI of the pelvis without and with MDC. Including soft parts, vascular district. The service replaces code 88.94.2	Diagnosis and monitoring	High diagnostics
89.01.4	Follow-up surgical visit	Diagnosis and monitoring	Monitoring
89.01.8	Follow-up endocrinological visit	Diagnosis and monitoring	Monitoring
89.01.A	Follow-up nuclear medicine visit	Radiotherapy	Radiotherapy
89.01.F	Oncology check-up visit	Diagnosis and monitoring	Monitoring
89.01.H	Check-up ENT visit	Diagnosis and monitoring	Monitoring
89.01.P	Follow-up radiotherapy visit	Radiotherapy	Radiotherapy
89.7B.3	First nuclear medicine visit	Radiotherapy	Radiotherapy
89.7C.1	First radiotherapy visit	Radiotherapy	Radiotherapy
90.05.1	Albumin test /serum /urine	Diagnosis and monitoring	Blood test
90.11.4	calcium test /serum /urine	Diagnosis and monitoring	Blood test
90.11.42	Total calcium [24h urine]	Diagnosis and monitoring	Blood test
90.11.5	Calcitonn	Diagnosis and monitoring	Blood test
90.24.3	Inorganic phosphate	Diagnosis and monitoring	Blood test
90.24.5	Phosphorus	Diagnosis and monitoring	Blood test
90.27.6	Placentar glycoprotein pregnancy specific - ioduria	Diagnosis and monitoring	Blood test
90.32.5	Total magnesium	Diagnosis and monitoring	Blood test
90.35.5	Parathyroid hormone (PTH)	Diagnosis and monitoring	Blood test
90.41.5	Thyroglobulin (Tg)	Diagnosis and monitoring	Blood test
90.42.1	Thyrotropin	Diagnosis and monitoring	Blood test
90.42.2	Thyrotropin (TSH): Serial dosages after TRH (4)	Diagnosis and monitoring	Blood test
90.42.3	Free thyroxine	Diagnosis and monitoring	Blood test
90.43.3	Free triodothyronine	Diagnosis and monitoring	Blood test
90.44.5	Vitamin D	Diagnosis and monitoring	Blood test
90.51.4	Antibodies to microsomes (AbTMS) or anti thyroperoxidase (AbTPO)	Diagnosis and monitoring	Blood test
90.54.4	Anti thyroglabulin antibodies (AbTg)	Diagnosis and monitoring	Blood test
90.56.3	Carcinoembryonic antigen (CEA)	Diagnosis and monitoring	Blood test

91.30.3	DNA segment analysis by sequencing	Diagnosis and monitoring	Diagnosis
91.36.5	DNA or RNA extraction	Diagnosis and monitoring	Diagnosis
91.39.1	Fine needle aspiration cytology NOS	Diagnosis and monitoring	Biopsy
91.48.1	ES. Histpcytopathological endocrine system: Thyroid needle biopsy	Diagnosis and monitoring	Biopsy
91.49.2	Venous blood test	Diagnosis and monitoring	Blood test
92.04.1	Sequential salivary gland scintigraphy with functional study	Diagnosis and monitoring	High diagnostics
92.16.1	Scan of lymphatic system	Diagnosis and monitoring	High diagnostics
92.18.2	Bone or articular scan	Diagnosis and monitoring	High diagnostics
92.18.3	Research for metastasis of thyroid tumours	Diagnosis and monitoring	High diagnostics
92.18.6	Whole body PET scan	Diagnosis and monitoring	High diagnostics
92.19.6	Segmental scintigraphy after whole body scintigraphy	Diagnosis and monitoring	High diagnostics
92.24.6	Teletherapy with linear accelerator with multiple fields or movement techniques with intensity modulation	Radiotherapy	Radiotherapy
92.24.7	Stereotactic radiotherapy first session or radiosurgery per session	Radiotherapy	Radiotherapy
92.29.5	Physical/dosimetric evaluation with computed tomography (CT) scans	Radiotherapy	Radiotherapy
92.29.7	Customized shielding	Radiotherapy	Radiotherapy
92.29.8	personalized system restraining	Radiotherapy	Radiotherapy
92.29.F	Definition of targets and critical organs with 3D techniques	Radiotherapy	Radiotherapy
93.71.8	Individual speech theraoy rehabilitation - The service replaces code. 93.71.1, 93.71.3 AND 93.72.1 (Re-education of communicative and/or cognitive disorders). Treatment of swallowing disorders with/without aids, phono-articular, functional or organic peripheral	Support therapy	Support therapy
99.24.1	Hormonal therapy	Support therapy	Support therapy

Table 4A. Thyroid cancer-related drugs (12+17) by AIC/ATC, description, category in Drug Prescription and Hospital Drugs databases (DP, HP).

Self-controlled crossover study in Epicost-2 project: 3,017 prevalent cases (diagnosed in 1/07/2015-31/12/2017; followed-up to 31/12/2018) and their 87,496 + 7,861 paired records collected in the DP, HP database.

DRUGS

**Database**	**ATC CODE**	**DESCRIPTION**	**CATEGORY**
**DP**	A07AA02	Nystatin	Support therapy
	A11CC04	Calcitriol	Support therapy
	A11CC05	Colecalciferol	Support therapy
	A12AA04	Calcium carbonate	Support therapy
	A12AA20	Calcium (different salts in combination)	Support therapy
	A12AX	Calcium, combinations with vitamin D and/or other drugs	Support therapy
	B01AB05	Enoxaparin	Antithrombotics
	B01AE07	Dabigatran etexilate	Antithrombotics
	H02AB07	Prednisone	Support therapy
	H03AA01	Levothyroxine sodium	Thyroid hormones
	H03AA02	Liothyronine sodium	Thyroid hormones
	J01CR02	Amoxicillin and beta-lactamase inhibitor	Antibiotics
	J01DD04	Ceftriaxone	Antibiotics
	J01MA02	Ciprofloxacin	Antibiotics
	L01XE12	Vandenatib	Chemotherapy
	N02AJ06	Codeine and paracetamol	Analgesics
	V04CJ01	Thyrotropin	Support therapy
**HD**	A11CC04	Thyrotropin	Support therapy
	A11CC05	Colecalciferol	Support therapy
	A11CC06	Calcifediol	Support therapy
	A12AA04	Calcium carbonate	Support therapy

	A12AA20	Calcium (different salts in combination)	Support therapy
	A12AX	Calcium, combinations with vitamin D and/or other drugs	Support therapy
	B01AB12	Bemiparin	Antithrombotics
	B03AB05	Ferric oxide polymaltose complexes	Support therapy
	H03AA01	Levothyroxine sodium	Thyroid hormones
	H03AA02	Liothyronine sodium	Thyroid hormones
	J01DD09	Cefodizime	Antibiotics
	J01DD16	Cefditoren	Antibiotics

Table 5A. Melanoma of the Skin-related diagnoses (63) by ICD9-CM, description, category, subcategory in Hospital Admission and Discharges database (HA).

Self-controlled crossover study in Epicost-2 project: 4,176 prevalent cases (diagnosed in 1/07/2015-31/12/2017; followed-up to 31/12/2018) and their 4,979 paired records collected in the HA.

DIAGNOSES

ICD9-CM CODE	DESCRIPTION	CATEGORY	SUBCATEGORY
035	Erysipelas	Diagnosis and monitoring	Diagnosis
038.8	Other specified sepsis	Diagnosis and monitoring	Diagnosis
041.04	Streptococcus infection in conditions classified elsewhere and of unspecified site, streptococcus, group D [Enterococcus]	Diagnosis and monitoring	Diagnosis
041.4	Escherichia coli NEC	Diagnosis and monitoring	Diagnosis
041.7	Pseudomonas infect NOS	Diagnosis and monitoring	Diagnosis
172.1	Malignant melanoma of unspecified eyelid, including canthus	Diagnosis and monitoring	Diagnosis
172.2	Malignant melanoma of skin of ear and external auditory cana	Diagnosis and monitoring	Diagnosis
172.3	Malignant melanoma of skin of other and unspecified parts of face	Diagnosis and monitoring	Diagnosis
172.4	Malignant melanoma of skin of scalp and neck	Diagnosis and monitoring	Diagnosis
172.5	Malignant melanoma of skin of trunk except scrotum	Diagnosis and monitoring	Diagnosis
172.6	Malignant melanoma of skin of upper limb, including shoulder	Diagnosis and monitoring	Diagnosis
172.7	Malignant melanoma of skin of lower limb, including hip	Diagnosis and monitoring	Diagnosis
172.8	Malignant melanoma of other specified sites of skin	Diagnosis and monitoring	Diagnosis
172.9	Melanoma of skin site unspecified	Diagnosis and monitoring	Diagnosis
196.0	Secondary and unspecified malignant neoplasm of lymph nodes of head face and neck	Diagnosis and monitoring	Diagnosis
196.1	Secondary and unspecified malignant neoplasm of intrathoracic lymph nodes	Diagnosis and monitoring	Diagnosis
196.2	Secondary and unspecified malignant neoplasm of intra-abdominal lymph nodes	Diagnosis and monitoring	Diagnosis
196.3	Secondary and unspecified malignant neoplasm of axilla and upper limb lymph nodes	Diagnosis and monitoring	Diagnosis
196.5	Secondary and unspecified malignant neoplasm of inguinal and lower limb lymph nodes	Diagnosis and monitoring	Diagnosis
196.6	Secondary and unspecified malignant neoplasm of intrapelvic lymph nodes	Diagnosis and monitoring	Diagnosis

196.9	Secondary and unspecified malignant neoplasm of lymph node, unspecified	Diagnosis and monitoring	Diagnosis
197.0	Secondary malignant neoplasm of lung	Diagnosis and monitoring	Diagnosis
197.1	Secondary malignant neoplasm of mediastinum	Diagnosis and monitoring	Diagnosis
197.7	Secondary Malignant Neoplasm of Liver	Diagnosis and monitoring	Diagnosis
198.2	Secondary malignant neoplasm of skin	Diagnosis and monitoring	Diagnosis
198.3	Secondary malignant neoplasm of brain and spinal cord	Diagnosis and monitoring	Diagnosis
198.89	Secondary malignant neoplasm of other specified sites	Diagnosis and monitoring	Diagnosis
238.2	Neoplasm of uncertain behavior of skin	Diagnosis and monitoring	Diagnosis
239.2	Neoplasm of unspecified nature of bone soft tissue and skin	Diagnosis and monitoring	Diagnosis
289.1	Chronic lymphadenitis	Diagnosis and monitoring	Diagnosis
289.3	Lymphadenitis unspecified except mesenteric	Diagnosis and monitoring	Diagnosis
362.56	Macular Pucker	Diagnosis and monitoring	Diagnosis
457.1	Other lymphedema	Diagnosis and monitoring	Diagnosis
457.2	Lymphangitis	Diagnosis and monitoring	Diagnosis
682.2	Cellulitis and abscess of trunk	Diagnosis and monitoring	Diagnosis
701.4	Keloid scar	Diagnosis and monitoring	Diagnosis
709.2	Scar conditions and fibrosis of skin	Diagnosis and monitoring	Diagnosis
709.4	Foreign body granuloma of the skin and subcutaneous tissue	Diagnosis and monitoring	Diagnosis
782.2	Localized superficial swelling mass or lump	Diagnosis and monitoring	Diagnosis
785.6	Enlargement of lymph nodes	Diagnosis and monitoring	Diagnosis
799.4	Cachexia	Diagnosis and monitoring	Diagnosis
995.91	Sepsis, unspecified organism	Diagnosis and monitoring	Diagnosis
V10.82	Special screening for malignant neoplasms of other sites	Diagnosis and monitoring	Diagnosis
V10.83	Incisional hernia without obstruction or gangrene	Diagnosis and monitoring	Diagnosis
V10.89	Hemorrhage complicating a procedure	Diagnosis and monitoring	Diagnosis
V64.3	Non-healing surgcl wound	Diagnosis and monitoring	Diagnosis
V70.7	Aftercare involving the use of plastic surgery	Diagnosis and monitoring	Diagnosis
V71.1	Other specified aftercare following surgery	Diagnosis and monitoring	Diagnosis
V72.6	Encounter for antineoplastic chemotherapy	Diagnosis and monitoring	Diagnosis

V76.49	Encounter for immunotherapy for neoplastic condition	Diagnosis and monitoring	Diagnosis
V58.11	Disrup-interrnal op wound	Chemotherapy	Chemotherapy
V58.12	Disrup-external op wound	Chemotherapy	Chemotherapy
V58.42	Postoperative wound infection	Chemotherapy	Chemotherapy
V51	Hematoma complic proc	Surgery	Plastic surgery
553.21	Aftercare following surgery for neoplasm	Surgery	Surgery
998.11	Personal history of malignant melanoma of skin	Surgery	Surgery
998.12	Personal history of other malignant neoplasm of skin	Surgery	Surgery
998.13	Personal history of malignant neoplasm of other sites	Surgery	Surgery
998.31	Other specified aftercare following surgery	Surgery	Surgery
998.32	Encounter for examination for normal comparison and control in clinical research program	Surgery	Surgery
998.59	Observation for suspected malignant neoplasm	Surgery	Surgery
998.83	Laboratory examination	Surgery	Surgery
V58.49	Seroma complicating a procedure	Surgery	Surgery

Table 5A. Melanoma of the Skin-related diagnoses (63) by ICD9-CM, description, category, subcategory in Hospital Admission and Discharges database (HA)

Self-controlled crossover study in Epicost-2 project: 4,176 prevalent cases (diagnosed in 1/07/2015-31/12/2017; followed-up to 31/12/2018)

and their 4,979 paired records collected in the HA

PROCEDURES

ICD9-CM CODE	DESCRIPTION	CATEGORY	SUBCATEGORY
035	Erysipelas	Diagnosis and monitoring	Diagnosis
038.8	Other specified sepsis	Diagnosis and monitoring	Diagnosis
041.04	Streptococcus infection in conditions classified elsewhere and of unspecified site, streptococcus, group D [Enterococcus]	Diagnosis and monitoring	Diagnosis
041.4	Escherichia coli NEC	Diagnosis and monitoring	Diagnosis
041.7	Pseudomonas infect NOS	Diagnosis and monitoring	Diagnosis
172.1	Malignant melanoma of unspecified eyelid, including canthus	Diagnosis and monitoring	Diagnosis
172.2	Malignant melanoma of skin of ear and external auditory cana	Diagnosis and monitoring	Diagnosis
172.3	Malignant melanoma of skin of other and unspecified parts of face	Diagnosis and monitoring	Diagnosis
172.4	Malignant melanoma of skin of scalp and neck	Diagnosis and monitoring	Diagnosis
172.5	Malignant melanoma of skin of trunk except scrotum	Diagnosis and monitoring	Diagnosis
172.6	Malignant melanoma of skin of upper limb, including shoulder	Diagnosis and monitoring	Diagnosis
172.7	Malignant melanoma of skin of lower limb, including hip	Diagnosis and monitoring	Diagnosis
172.8	Malignant melanoma of other specified sites of skin	Diagnosis and monitoring	Diagnosis
172.9	Melanoma of skin site unspecified	Diagnosis and monitoring	Diagnosis
196.0	Secondary and unspecified malignant neoplasm of lymph nodes of head face and neck	Diagnosis and monitoring	Diagnosis
196.1	Secondary and unspecified malignant neoplasm of intrathoracic lymph nodes	Diagnosis and monitoring	Diagnosis
196.2	Secondary and unspecified malignant neoplasm of intra-abdominal lymph	Diagnosis and monitoring	Diagnosis

	nodes		
196.3	Secondary and unspecified malignant neoplasm of axilla and upper limb lymph nodes	Diagnosis and monitoring	Diagnosis
196.5	Secondary and unspecified malignant neoplasm of inguinal and lower limb lymph nodes	Diagnosis and monitoring	Diagnosis
196.6	Secondary and unspecified malignant neoplasm of intrapelvic lymph nodes	Diagnosis and monitoring	Diagnosis
196.9	Secondary and unspecified malignant neoplasm of lymph node, unspecified	Diagnosis and monitoring	Diagnosis
197.0	Secondary malignant neoplasm of lung	Diagnosis and monitoring	Diagnosis
197.1	Secondary malignant neoplasm of mediastinum	Diagnosis and monitoring	Diagnosis
197.7	Secondary Malignant Neoplasm of Liver	Diagnosis and monitoring	Diagnosis
198.2	Secondary malignant neoplasm of skin	Diagnosis and monitoring	Diagnosis
198.3	Secondary malignant neoplasm of brain and spinal cord	Diagnosis and monitoring	Diagnosis
198.89	Secondary malignant neoplasm of other specified sites	Diagnosis and monitoring	Diagnosis
238.2	Neoplasm of uncertain behavior of skin	Diagnosis and monitoring	Diagnosis
239.2	Neoplasm of unspecified nature of bone soft tissue and skin	Diagnosis and monitoring	Diagnosis
289.1	Chronic lymphadenitis	Diagnosis and monitoring	Diagnosis
289.3	Lymphadenitis unspecified except mesenteric	Diagnosis and monitoring	Diagnosis
362.56	Macular Pucker	Diagnosis and monitoring	Diagnosis
457.1	Other lymphedema	Diagnosis and monitoring	Diagnosis
457.2	Lymphangitis	Diagnosis and monitoring	Diagnosis
682.2	Cellulitis and abscess of trunk	Diagnosis and monitoring	Diagnosis
701.4	Keloid scar	Diagnosis and monitoring	Diagnosis
709.2	Scar conditions and fibrosis of skin	Diagnosis and monitoring	Diagnosis
709.4	Foreign body granuloma of the skin and subcutaneous tissue	Diagnosis and monitoring	Diagnosis
782.2	Localized superficial swelling mass or lump	Diagnosis and monitoring	Diagnosis
785.6	Enlargement of lymph nodes	Diagnosis and monitoring	Diagnosis
799.4	Cachexia	Diagnosis and monitoring	Diagnosis
995.91	Sepsis, unspecified organism	Diagnosis and monitoring	Diagnosis
V10.82	Special screening for malignant neoplasms of other sites	Diagnosis and monitoring	Diagnosis
V10.83	Incisional hernia without obstruction or gangrene	Diagnosis and monitoring	Diagnosis

V10.89	Hemorrhage complicating a procedure	Diagnosis and monitoring	Diagnosis
V64.3	Non-healing surgcl wound	Diagnosis and monitoring	Diagnosis
V70.7	Aftercare involving the use of plastic surgery	Diagnosis and monitoring	Diagnosis
V71.1	Other specified aftercare following surgery	Diagnosis and monitoring	Diagnosis
V72.6	Encounter for antineoplastic chemotherapy	Diagnosis and monitoring	Diagnosis
V76.49	Encounter for immunotherapy for neoplastic condition	Diagnosis and monitoring	Diagnosis
V58.11	Disrup-interrnal op wound	Chemotherapy	Chemotherapy
V58.12	Disrup-external op wound	Chemotherapy	Chemotherapy
V58.42	Postoperative wound infection	Chemotherapy	Chemotherapy
V51	Hematoma complic proc	Surgery	Plastic surgery
553.21	Aftercare following surgery for neoplasm	Surgery	Surgery
998.11	Personal history of malignant melanoma of skin	Surgery	Surgery
998.12	Personal history of other malignant neoplasm of skin	Surgery	Surgery
998.13	Personal history of malignant neoplasm of other sites	Surgery	Surgery
998.31	Other specified aftercare following surgery	Surgery	Surgery
998.32	Encounter for examination for normal comparison and control in clinical research program	Surgery	Surgery
998.59	Observation for suspected malignant neoplasm	Surgery	Surgery
998.83	Laboratory examination	Surgery	Surgery
V58.49	Seroma complicating a procedure	Surgery	Surgery

Table 6A. Melanoma of the Skinj-related procedures (108) by ICD9-CM, description, category,subcategory in Hospital Admissions and database (HA).
Self-controlled crossover study in Epicost-2 project: 4,176 prevalent cases (diagnosed in 1/07/2015-31/12/2017; followed-up to 31/12/2018) and their 4,979 paired records collected in the HA.

PROCEDURES

ICD9-CM CODE	DESCRIPTION	CATEGORY	SUBCATEGORY
01.59	Other excision or destruction of lesion or tissue of brain	Surgery	Surgery
08.23	Excision of major lesion of eyelid, partial-thickness	Surgery	Surgery
08.61	Reconstruction of eyelid with skin flap or graft	Surgery	Plastic Surgery
08.69	Other reconstruction of eyelid with flaps or grafts	Surgery	Plastic Surgery
08.70	Reconstruction of eyelid, not otherwise specified	Surgery	Plastic Surgery
14.9	Other operations on retina, choroid, and posterior chamber	Surgery	Surgery
18.29	Excision or destruction of other lesion of external ear	Surgery	Surgery
18.31	Radical excision of lesion of external ear	Surgery	Surgery
18.39	Other excision of external ear	Surgery	Surgery
18.71	Construction of auricle of ear	Surgery	Plastic Surgery
18.79	Other plastic repair of external ear	Surgery	Plastic Surgery
21.86	Limited rhinoplasty	Surgery	Plastic Surgery
26.31	Partial sialoadenectomy	Surgery	Surgery
26.32	Complete sialoadenectomy	Surgery	Surgery
26.99	Other operations on salivary gland or duct	Surgery	Surgery
38.89	Other surgical occlusion of vessels, lower limb veins	Surgery	Surgery
40.11	Biopsy of lymphatic structure	Surgery	Surgery
40.19	Other diagnostic procedures on lymphatic structures	Diagnosis and monitoring	Biopsy
40.21	Excision of deep cervical lymph node	Surgery	Lymphadenectomy
40.23	Excision of axillary lymph node	Surgery	Lymphadenectomy

40.24	Excision of inguinal lymph node	Surgery	Lymphadenectomy
40.29	Simple excision of other lymphatic structure	Surgery	Lymphadenectomy
40.3	Regional lymph node excision	Surgery	Lymphadenectomy
40.41	Radical neck dissection, unilateral	Surgery	Lymphadenectomy
40.50	Radical excision of lymph nodes, not otherwise specified	Surgery	Lymphadenectomy
40.51	Radical excision of axillary lymph nodes	Diagnosis and monitoring	Diagnosis and monitoring
40.53	Radical excision of iliac lymph nodes	Surgery	Lymphadenectomy
40.54	Radical groin dissection	Surgery	Lymphadenectomy
40.59	Radical excision of other lymph nodes	Surgery	Lymphadenectomy
50.11	Closed (percutaneous) [needle] biopsy of liver	Diagnosis and monitoring	Biopsy
83.21	Open biopsy of soft tissue	Diagnosis and monitoring	Biopsy
83.32	Excision of lesion of muscle	Surgery	Plastic Surgery
83.39	Excision of lesion of other soft tissue	Surgery	Plastic Surgery
83.49	Other excision of soft tissue	Surgery	Plastic Surgery
84.01	Amputation and disarticulation of finger	Surgery	Plastic Surgery
84.02	Amputation and disarticulation of thumb	Surgery	Plastic Surgery
84.11	Amputation of toe	Surgery	Plastic Surgery
86.01	Aspiration of skin and subcutaneous tissue	Surgery	Plastic Surgery
86.04	Other incision with drainage of skin and subcutaneous tissue	Surgery	Plastic Surgery
86.09	Other incision of skin and subcutaneous tissue	Surgery	Plastic Surgery
86.11	Closed biopsy of skin and subcutaneous tissue	Diagnosis and monitoring	Biopsy
86.19	Other diagnostic procedures on skin and subcutaneous tissue	Diagnosis and monitoring	Other diagnostic procedure
86.22	Excisional debridement of wound, infection, or burn	Surgery	Plastic Surgery
86.23	Removal of nail, nail bed, or nail fold	Surgery	Plastic Surgery
86.3	Other local excision or destruction of lesion or tissue of skin and subcutaneous tissue	Surgery	Plastic Surgery
86.4	Radical excision of skin lesion	Surgery	Surgery
86.59	Closure of skin and subcutaneous tissue of other sites	Surgery	Other surgery
86.60	Free skin graft, not otherwise specified	Surgery	Plastic Surgery

86.63	Full-thickness skin graft to other sites	Surgery	Plastic Surgery
86.66	Homograft to skin	Surgery	Plastic Surgery
86.67	Dermal regenerative graft	Surgery	Plastic Surgery
86.69	Other skin graft to other sites	Surgery	Plastic Surgery
86.70	Pedicle or flap graft, not otherwise specified	Surgery	Plastic Surgery
86.71	Cutting and preparation of pedicle grafts or flaps	Surgery	Plastic Surgery
86.72	Advancement of pedicle graft	Surgery	Plastic Surgery
86.74	Attachment of pedicle or flap graft to other sites	Surgery	Plastic Surgery
86.75	Revision of pedicle or flap graft	Surgery	Plastic Surgery
86.82	Facial rhytidectomy	Surgery	Plastic Surgery
86.89	Other repair and reconstruction of skin and subcutaneous tissue	Surgery	Plastic Surgery
86.91	Excision of skin for graft	Surgery	Plastic Surgery
86.99	Other operations on skin and subcutaneous tissue	Surgery	Plastic Surgery
87.03	Computerized axial tomography of head	Diagnosis and monitoring	High diagnostic
87.41	Computerized axial tomography of thorax	Diagnosis and monitoring	High diagnostic
87.44	Routine chest x-ray, so described	Diagnosis and monitoring	Conventional radiology
88.01	Computerized axial tomography of abdomen	Diagnosis and monitoring	High diagnostic
88.38	Other computerized axial tomography	Diagnosis and monitoring	High diagnostic
88.72	Diagnostic ultrasound of heart	Diagnosis and monitoring	Cardiologic assessment
88.76	Diagnostic ultrasound of abdomen and retroperitoneum	Diagnosis and monitoring	Ultrasonography
88.79	Other diagnostic ultrasound	Diagnosis and monitoring	Ultrasonography
88.90	Diagnostic imaging, not elsewhere classified	Diagnosis and monitoring	Other diagnostic procedure
88.91	Magnetic resonance imaging of brain and brain stem	Diagnosis and monitoring	High diagnostic
88.97	Magnetic resonance imaging of other and unspecified sites	Diagnosis and monitoring	High diagnostic
89.01	Interview and evaluation, described as brief	Diagnosis and monitoring	Monitoring
89.02	Interview and evaluation, described as limited	Diagnosis and monitoring	Monitoring
89.03	Interview and evaluation, described as comprehensive	Diagnosis and monitoring	Monitoring
89.05	Diagnostic interview and evaluation, not otherwise specified	Diagnosis and monitoring	Monitoring
89.06	Consultation, described as limited	Diagnosis and monitoring	Monitoring

89.07	Consultation, described as comprehensive	Diagnosis and monitoring	Monitoring
89.08	Other consultation	Diagnosis and monitoring	Monitoring
89.09	Consultation, not otherwise specified	Diagnosis and monitoring	Monitoring
89.52	Electrocardiogram	Diagnosis and monitoring	Cardiologic assessment
89.54	Electrographic monitoring	Diagnosis and monitoring	Cardiologic assessment
89.7	General physical examination	Diagnosis and monitoring	Monitoring
90.39	Microscopic examination of specimen from ear, nose, throat, and larynx, other microscopic examination	Diagnosis and monitoring	Other diagnostic procedure
90.52	Microscopic examination of blood, culture	Diagnosis and monitoring	Other diagnostic procedure
90.59	Microscopic examination of blood, other microscopic examination	Diagnosis and monitoring	Other diagnostic procedure
90.79	Microscopic examination of specimen from lymph node and of lymph, other microscopic examination	Diagnosis and monitoring	Other diagnostic procedure
91.69	Microscopic examination of specimen from skin and other integument, other microscopic examination	Diagnosis and monitoring	Other diagnostic procedure
91.79	Microscopic examination of specimen from operative wound, other microscopic examination	Diagnosis and monitoring	Other diagnostic procedure
91.99	Microscopic examination of specimen from unspecified site, other microscopic examination	Diagnosis and monitoring	Other diagnostic procedure
92.16	Scan of lymphatic system	Diagnosis and monitoring	High diagnostic
92.18	Total body scan	Diagnosis and monitoring	High diagnostic
92.19	Scan of other sites	Diagnosis and monitoring	High diagnostic
92.28	Injection or instillation of radioisotopes	Radiotherapy	Radiotherapy
92.29	Other radiotherapeutic procedure	Radiotherapy	Radiotherapy
92.31	Single source photon radiosurgery	Radiotherapy	Radiotherapy
93.56	Application of pressure dressing	Support therapy	Support therapy
93.57	Application of other wound dressing	Support therapy	Support therapy
93.59	Other immobilization, pressure, and attention to wound	Support therapy	Support therapy
94.49	Other counseling	Diagnosis and monitoring	Monitoring
96.59	Other irrigation of wound	Support therapy	Support therapy

99.04	Transfusion of packed cells	Transfusion	Transfusion
99.21	Injection of antibiotic	Support therapy	Support therapy
99.25	Injection or infusion of cancer chemotherapeutic substance	Chemotherapy	Chemotherapy
99.28	Injection or infusion of biological response modifier [BRM] as an antineoplastic agent	Biologic therapy	Biologic therapy
99.29	Injection or infusion of other therapeutic or prophylactic substance	Support therapy	Support therapy
99.85	Hyperthermia for treatment of cancer	Radiotherapy	Radiotherapy
99.99	Other miscellaneous procedures	Other	Other

Table 7A. Melanoma of the Skin-related procedures (75) by ICD9-CM, description, category, subcategory in Outpatients Services database (OPS).
Self-controlled crossover study in Epicost-2 project: 4,176 prevalent cases (diagnosed in 1/07/2015-31/12/2017; followed-up to 31/12/2018) and their 272,215 paired records collected in the OPS database.

PROCEDURES

ICD9-CM CODE	DESCRIPTION	CATEGORY	SUBCATEGORY
08.23	Excision of major lesion of eyelid, partial-thickness	Surgery	Surgery
26.11	Closed [needle] biopsy of salivary gland or duct	Diagnosis and monitoring	Biopsy
40.11	Open biopsy of lymphatic structures: cervical, supraclavicular or supraclavicular lymph nodes; axillary lymph nodes	Diagnosis and monitoring	Biopsy
04.81.1	Injection of anesthetic into peripheral nerve for analgesia. gasserian ganglion and branches block.<br>Including drug. Excluded: anesthesia for surgery.<br>Included: possible ultrasound guide.	Support therapy	Support therapy
52.13	Endoscopic retrograde pancreatography [ERP]	Diagnosis and monitoring	Endoscopic Procedure
86.01	Aspiration of skin and subcutaneous tissue;	Diagnosis and monitoring	Biopsy
86.60	Free skin graft, NOS	Plastic surgery	Plastic surgery
86.74	Attachment of pedicle or flap graft to other sites	Plastic surgery	Plastic surgery
86.84	Relaxation of scar or web contracture of skin	Plastic surgery	Plastic surgery
89.02	Interview and evaluation, described as limited	Diagnosis and monitoring	Specialist examination
89.13	Neurologic examination	Diagnosis and monitoring	Specialist examination
93.03	Prosthetic evaluation	Diagnosis and monitoring	Specialist examination
38.59.1	Ligation and stripping of varicose veins, lower limb veins	Surgery	Surgery
38.59.2	Ligation and stripping of varicose veins, lower limb veins	Surgery	Surgery
38.99.1	Contrast material administration for MRI radiotherapy simulation	Radiotherapy	Radiotherapy
50.19.1	[Percutaneous] [needle] biopsy of liver	Diagnosis and monitoring	Biopsy
54.24.1	Closed [percutaneous] [needle] biopsy of intra-abdominal mass	Diagnosis and monitoring	Biopsy
87.03.3	Computerized axial tomography of head	Diagnosis and monitoring	High diagnostics
87.03.7	Computerized axial tomography (CAT) of neck	Diagnosis and monitoring	High diagnostics
88.01.4	Computed tomography (CAT) of lower abdomen, without and	Diagnosis and monitoring	High diagnostics

	with contrast.		
88.41.3	Arteriography of cerebral arteries	Diagnosis and monitoring	High diagnostics
88.47.2	Arteriography of other intra-abdominal arteries	Diagnosis and monitoring	High diagnostics
88.77.2	Diagnostic ultrasound of peripheral vascular system	Diagnosis and monitoring	Ultrasonography
88.90.2	Three-dimensional computerized Axial tomography (CAT) reconstruction	Diagnosis and monitoring	High diagnostics
88.91.4	Magnetic resonance imaging of brain and brain stem	Diagnosis and monitoring	High diagnostics
88.91.7	Magnetic resonance imaging of brain and brain stem	Diagnosis and monitoring	High diagnostics
88.92.1	Magnetic resonance imaging (MRI) of Thorax, without and with contrast	Diagnosis and monitoring	High diagnostics
88.92.4	Magnetic resonance imaging of chest and myocardium	Diagnosis and monitoring	High diagnostics
88.93.1	Magnetic resonance imaging (MRI) of vertebral column, without and with contrast	Diagnosis and monitoring	High diagnostics
88.95.5	Magnetic resonance imaging (MRI) of lower abdomen and pelvic cavity, without and with contrast	Diagnosis and monitoring	High diagnostics
88.96.1	Other intraoperative magnetic resonance imaging	Diagnosis and monitoring	High diagnostics
88.96.2	Other intraoperative magnetic resonance imaging	Diagnosis and monitoring	High diagnostics
89.01.6	Interview and evaluation, described as brief	Diagnosis and monitoring	Specialist examination
90.01.5	Microscopic examination of specimen from nervous system and of spinal fluid, bacterial smear (BILE ACIDS)	Diagnosis and monitoring	Blood test
90.05.5	Alpha 1 fetoprotein [S/La/Alb]	Diagnosis and monitoring	Bio-marker
90.16.7	Microscopic examination of specimen from endocrine gland, not elsewhere classified, cell block and Papanicolaou smear	Diagnosis and monitoring	Blood test
90.18.4	Neuron specific enolasis (NSE)	Diagnosis and monitoring	Bio-marker
90.36.6	Microscopic examination of specimen from ear, nose, throat, and larynx, cell block and Papanicolaou smear	Diagnosis and monitoring	Blood test
90.55.1	Cancer Antigen 125	Diagnosis and monitoring	Genetic marker
90.70.1	INTERFERON.	Diagnosis and monitoring	Blood test
90.84.3	Microscopic examination of specimen from upper gastrointestinal tract and of vomitus, parasitology	Diagnosis and monitoring	Culture test
90.93.3	Microscopic examination of specimen from lower gastrointestinal tract and of stool, culture and sensitivity	Diagnosis and monitoring	Culture test

90.98.4	FYRMUS RESEARCH IN DIFFERENT BIOLOGICAL MATERIALS. Including: microscopic examination and culture examination. If<br><br>positive, including identification	Diagnosis and monitoring	Culture test
90.98.5	MYCETES [FILAMENTOUS FYNCES] MICROSCOPIC RESEARCH IN DIFFERENT BIOLOGICAL MATERIALS NAS.	Diagnosis and monitoring	Culture test
91.36.1	Storage of DNA or RNA sample	Diagnosis and monitoring	Genetic test
91.37.3	Hybridization in situ (FISH)	Diagnosis and monitoring	Genetic test
91.39.3	Cytological examination of effusions	Diagnosis and monitoring	Biopsy
91.39.6	Immuno-histochemical predictive marker of response to therapy; HERCEP-TEST (EGFR)	Diagnosis and monitoring	Biopsy
91.41.1	Incisional biopsy (Skin and/or soft tissue)	Diagnosis and monitoring	Biopsy
91.41.2	Fine needle biopsy of liver	Diagnosis and monitoring	Biopsy
92.11.7	Cerebral scan	Diagnosis and monitoring	High diagnostics
92.12.9	Scan of other sites of head	Diagnosis and monitoring	High diagnostics
92.19.6	Segmental scintigraphy after total body scintigraphy	Diagnosis and monitoring	High diagnostics
92.19.8	Total body positron emission tomography (PET) with computerized Axial tomography (CAT)	Diagnosis and monitoring	High diagnostics
92.29.3	Target acquisition via magnetic resonance (MR)	Radiotherapy	Radiotherapy
92.29.6	In vivo dosimetry	Radiotherapy	Radiotherapy
93.04.2	Manual testing of muscle function; segmental test on joints and muscles	Diagnosis and monitoring	Physiatry
93.05.1	Range of motion testing	Diagnosis and monitoring	Physiatry
93.11.2	Individual (motor)rehabilitation for seriously motion-impaired patient; simple instrumental rehabilitation	Diagnosis and monitoring	Physiatry
93.31.2	Assisted exercise in pool	Diagnosis and monitoring	Physiatry
93.39.2	Massotherapy for lymphatic drainage	Diagnosis and monitoring	Physiatry
88.72.11	Diagnostic ultrasound of heart	Diagnosis and monitoring	Ultrasonography
88.77.11	Diagnostic ultrasound of peripheral vascular system	Diagnosis and monitoring	Ultrasonography
91.40.52	ES. HISTOCYTOPATHOLOGICAL SKIN AND/OR SOFT	Diagnosis and monitoring	Biopsy

	TISSUES: Multiple excisional biopsies Not associated with code 91.40.51		
92.18.61	Total body scan	Diagnosis and monitoring	High diagnostics
88.79.H	Other diagnostic ultrasound	Diagnosis and monitoring	Ultrasonography
88.79.J	Other diagnostic ultrasound	Diagnosis and monitoring	High diagnostics
88.91.N	Magnetic resonance imaging of brain and brain stem	Diagnosis and monitoring	High diagnostics
88.97.A	Magnetic resonance imaging of other and unspecified sites	Diagnosis and monitoring	High diagnostics
89.01.N	Interview and evaluation, described as brief	Diagnosis and monitoring	Specialist examination
89.7A.6	FIRST VASCULAR SURGICAL VISIT	Diagnosis and monitoring	Specialist examination
89.7B.9	FIRST ENT VISIT. Included, based on the specific clinical problem: possible otomicroscopy, vestibular function examination, use of optical fibers, removal of earwax	Diagnosis and monitoring	Specialist examination
90.38.Z	PROCALCITONIN.	Diagnosis and monitoring	Blood test
91.40.G	EX. HISTOPATHOLOGICAL SKIN AND/OR SOFT TISSUES. With biopsy or excision of neoformation. Included:<br><br>any additional histochemical and/or immunohistochemical analyzes necessary to complete the<br><br>diagnosis. For sample	Diagnosis and monitoring	Biopsy
92.29.K	Other radiotherapeutic procedure	Radiotherapy	Radiotherapy

Table 8A. Melanoma of the Skin-related drug (54+33) by AIC/ATC, description, category in Drug Prescription and Hospital Drugs databases (DP, HP). Self-controlled crossover study in Epicost-2 project: 4,176 prevalent cases (diagnosed in 1/07/2015-31/12/2017; followed-up to 31/12/2018) and their 112,910+13,908 paired records collected in the DP, HP databases.

DRUGS

Database	ATC CODE	DESCRIPTION	CATEGORY
**DP**	A02BA02	Ranitidine	Support therapy
	A07AA02	Nystatin	Support therapy
	B01AB05	Enoxaparin	Antithrombotics
	B01AB06	Nadroparin	Antithrombotics
	B01AB07	Parnaparin	Antithrombotics
	B01AC04	Clopidogrel	Antithrombotics
	B01AF01	Rivaroxaban	Antithrombotics
	B01AX05	Fondaparinux	Antithrombotics
	B02AA02	Tranexamic acid	Support therapy
	H02AB07	Prednisone	Cortisone
	J01AA02	Doxycycline	Antibiotics
	J01CA04	Amoxicillin	Antibiotics
	J01CR02	Amoxicillin and beta-lactamase inhibitor	Antibiotics
	J01FA09	Clarithromycin	Antibiotics
	J01MA02	Ciprofloxacin	Antibiotics
	J01MA12	Levofloxacin	Antibiotics
	L01AX03	Temozolomide	Chemotherapic drug
	L01XC17	Nivolumab	Biologic therapy
	L01XC18	Pembrolizumab	Biologic therapy
	L01XE23	Dabrafenib	Biologic therapy
	L01XE25	Trametinib	Biologic therapy
	L03AB04	Interferon alfa-2a	Chemotherapic drug

	L03AB05	Interferon alfa-2b	Chemotherapic drug
	L04AD02	Tacrolimus	Support therapy
	M01AB01	Indometacin	Analgesics
	M01AE03	Ketoprofen	Analgesics
	M04AA01	Allopurinol	Support therapy
	N02AJ06	Codeine and paracetamol	Analgesics
	N02BE01	Paracetamol	Analgesics
	N03AX12	Gabapentin	Analgesics
	N05AH03	Olanzapine	Support therapy
	N06AX21	Duloxetine	Analgesics
	P01AB01	Metronidazole	Antibiotics
**HD**	A02BA02	Ranitidine	Support therapy
	A02BC01	Omeprazole	Support therapy
	A02BC02	Pantoprazole	Support therapy
	A03FA01	Metoclopramide	Support therapy
	A04AA01	Ondansetron	Support therapy
	B01AB05	Enoxaparin	Antithrombotics
	B01AC06	Acetylsalicylic acid	Antithrombotics
	B01AC30	Platelet aggregation inhibitor combinations excl. heparin	Antithrombotics
	D05AX52	Calcipotriol, combinations	Support therapy
	D11AX18	Diclofenac	Analgesics
	H02AB02	Dexamethasone	Cortisone
	H02AB04	Methylprednisolone	Cortisone
	H02AB07	Prednisone	Cortisone
	H02AB10	Cortisone	Cortisone
	J01CA04	Amoxicillin	Antibiotics
	J01CR02	Amoxicillin and beta-lactamase inhibitor	Antibiotics
	J01DB01	Cefalexin	Antibiotics
	J01DD08	Cefixime	Antibiotics

	J01DD16	Cefditoren	Antibiotics
	J01MA12	Levofloxacin	Antibiotics
	L03AB04	Interferon alfa-2a	Chemotherapic drug
	L03AB05	Interferon alfa-2b	Chemotherapic drug
	M01AB05	Diclofenac	Analgesics
	M01AC02	Tenoxicam	Analgesics
	N01BB02	Lidocaine	Analgesics
	N02AA55	Oxycodone and naloxone	Analgesics
	N02AB03	Fentanyl	Analgesics
	N02AJ06	Codeine and paracetamol	Analgesics
	N02AX06	Tapentadol	Analgesics
	N02BE01	Paracetamol	Analgesics
	N03AX16	Pregabalin	Analgesics
	N06AB10	Escitalopram	Antidepressant
	N06AX11	Mirtazapine	Antidepressant
	N06AX21	Duloxetine	Analgesics
	N06AX26	Vortioxetine	Antidepressant
	R03AK07	Formoterol and budesonide	Antiasthmatic
	R06AE07	Cetirizine	Antihistamine
	R06AX27	Desloratadine	Antihistamine
	S01EC03	Dorzolamide	Glaucoma medication
	S01ED05	Carteolol	Glaucoma medication
	S01ED51	Timolol, combinations	Glaucoma medication
