# Supplementary material for: An indirect approach to identify the healthcare services for thyroid and melanoma cancer patients in Italy: Epicost-2 project
Source: Tumori. 2025 Sep 4;111(5):400–12. doi: 10.1177/03008916251353109 (PMC12476483; doi:10.1177/03008916251353109)
Supplement: sj-pdf-2-tmj-10.1177_03008916251353109 – Supplemental material for An indirect approach to identify the healthcare services for thyroid and melanoma cancer patients in Italy: Epicost-2 project [file sj-pdf-2-tmj-10.1177_03008916251353109.pdf]

Table 1B. Top 10 most frequent cancer-related procedures for Thyroid cancer by International statistical classification of disease (ICD9-CM), description, category, subcategory in Hospital Admission and Discharge database (HA) and phase of care. Epicost-2 prevalence population of 12,157 TC patients.

Phase of care	ICD9-CM code	Description	Category	Subcategory	N	%
**Initial**	06.4	*Complete thyroidectomy*	Surgery	Total Thyroidectomy	1.091	37,1
	92,29	*Other radiotherapeutic procedure*	Radiotherapy	Radiotherapy	376	12,8
	92,18	*Total body scan*	Diagnosis and monitoring	High diagnostic	316	10,8
	40,21	*Excision of deep cervical lymph node*	Surgery	Lymphadenectomy	262	8,9
	92,28	*Injection or instillation of radioisotopes*	Radiotherapy	Radiotherapy	199	6,8
	06.2	*Unilateral thyroid lobectomy*	Surgery	Partial Thyroidectomy	106	3,6
	99,29	*Injection or infusion of other therapeutic or prophylactic substance*	Support therapy	Support therapy	103	3,5
	40,41	*Radical neck dissection, unilateral*	Surgery	Lymphadenectomy	55	1,9
	92,01	*Thyroid scan and radioisotope function studies*	Diagnosis and monitoring	High diagnostic	52	1,8
	88,71	*Diagnostic ultrasound of head and neck*	Diagnosis and monitoring	Diagnosis	45	1,5
**Total**					**2.937**	**100,0**
**Continuing**	06.4	*Complete thyroidectomy*	Surgery	Total Thyroidectomy	3.563	29,9
	92,29	*Other radiotherapeutic procedure*	Radiotherapy	Radiotherapy	1.909	16,0
	92,18	*Total body scan*	Diagnosis and monitoring	High diagnostic	1.564	13,1
	40,21	*Excision of deep cervical lymph node*	Surgery	Lymphadenectomy	991	8,3
	99,29	*Injection or infusion of other therapeutic or prophylactic substance*	Support therapy	Support therapy	644	5,4
	92,28	*Injection or instillation of radioisotopes*	Radiotherapy	Radiotherapy	569	4,8
	06.2	*Unilateral thyroid lobectomy*	Surgery	Partial Thyroidectomy	352	2,9
	88,71	*Diagnostic ultrasound of head and neck*	Diagnosis and monitoring	Diagnosis	347	2,9
	87,03	*Computerized axial tomography of head*	Diagnosis and monitoring	High diagnostic	206	1,7
	40,41	*Radical neck dissection, unilateral*	Surgery	Lymphadenectomy	202	1,7
**Total**					**11.933**	**100,0**
**Final**	99,29	*Injection or infusion of other therapeutic or prophylactic substance*	Support therapy	Support therapy	34	17,3
	87,03	*Computerized axial tomography of head*	Diagnosis and monitoring	High diagnostic	24	12,2
	06.4	*Complete thyroidectomy*	Surgery	Total Thyroidectomy	21	10,7
	40,21	*Excision of deep cervical lymph node*	Surgery	Lymphadenectomy	13	6,6
	92,18	*Total body scan*	Diagnosis and monitoring	High diagnostic	12	6,1

	92,29	*Other radiotherapeutic procedure*	Radiotherapy	Radiotherapy	12	6,1
	87,41	*Computerized axial tomography of thorax*	Diagnosis and monitoring	High diagnostic	11	5,6
	92,28	*Injection or instillation of radioisotopes*	Radiotherapy	Radiotherapy	10	5,1
	40,41	*Radical neck dissection, unilateral*	Surgery	Lymphadenectomy	8	4,1
	99,23	*Injection Of Steroid*	Support therapy	Support therapy	7	3,6
**Total**					**196**	**100,0**

Table 2B. Top 10 most cancer-related procedures for Thyroid cancer by International statistical classification of disease (ICD9-CM) description, category, subcategory in Outpatients Services database (OPS) and phase of care. Epicost-2 prevalence

population of 12,157 TC patients.

Phase of care	ICD9-CM code	Description	Category	Subcategory	N	%
**Initial**	91.49.2	*Venous blood test*	Diagnosis and monitoring	Blood test	14.991	20,2
	90.42.1	*Thyrotropin*	Diagnosis and monitoring	Blood test	10.017	13,5
	90.42.3	*Free thyroxine*	Diagnosis and monitoring	Blood test	8.051	10,8
	90.43.3	*Free triodothyronine*	Diagnosis and monitoring	Blood test	5.046	6,8
	90.11.4	*calcium test /serum /urine*	Diagnosis and monitoring	Blood test	5.001	6,7
	90.41.5	*Thyroglobulin (Tg)*	Diagnosis and monitoring	Blood test	4.278	5,8
	90.54.4	*Anti thyroglobulin antibodies (AbTg)*	Diagnosis and monitoring	Blood test	3.987	5,4
	88.71.4	*Ultrasound diagnostics of the head and neck*	Diagnosis and monitoring	Diagnosis	2.839	3,8
	90.44.5	*Vitamin D*	Diagnosis and monitoring	Blood test	2.570	3,5
	89.01.8	*Follow-up endocrinological visit*	Diagnosis and monitoring	Monitoring	2.462	3,3
**Total**					**74.388**	**100,0**
**Continuing**	91.49.2	*Venous blood test*	Diagnosis and monitoring	Blood test	140.219	18,7
	90.42.1	*Thyrotropin*	Diagnosis and monitoring	Blood test	103.103	13,8
	90.42.3	*Free thyroxine*	Diagnosis and monitoring	Blood test	86.085	11,5
	90.41.5	*Thyroglobulin (Tg)*	Diagnosis and monitoring	Blood test	56.688	7,6
	90.43.3	*Free triodothyronine*	Diagnosis and monitoring	Blood test	56.676	7,6
	90.11.4	*calcium test /serum /urine*	Diagnosis and monitoring	Blood test	54.467	7,3
	90.54.4	*Anti thyroglobulin antibodies (AbTg)*	Diagnosis and monitoring	Blood test	48.193	6,4
	88.71.4	*Ultrasound diagnostics of the head and neck*	Diagnosis and monitoring	Diagnosis	32.869	4,4
	90.44.5	*Vitamin D*	Diagnosis and monitoring	Blood test	32.823	4,4
	89.01.8	*Follow-up endocrinological visit*	Diagnosis and monitoring	Monitoring	22.286	3,0
**Total**					**749.721**	**100,0**
**Final**	91.49.2	*Venous blood test*	Diagnosis and monitoring	Blood test	1.878	26,2
	90.42.1	*Thyrotropin*	Diagnosis and monitoring	Blood test	806	11,2
	90.11.4	*calcium test /serum /urine*	Diagnosis and monitoring	Blood test	628	8,7
	90.42.3	*Free thyroxine*	Diagnosis and monitoring	Blood test	586	8,2
	90.43.3	*Free triodothyronine*	Diagnosis and monitoring	Blood test	374	5,2
	90.41.5	*Thyroglobulin (Tg)*	Diagnosis and monitoring	Blood test	307	4,3
	90.54.4	*Anti thyroglobulin antibodies (AbTg)*	Diagnosis and monitoring	Blood test	266	3,7
	90.44.5	*Vitamin D*	Diagnosis and monitoring	Blood test	209	2,9
	90.05.1	*Albumin test /serum /urine*	Diagnosis and monitoring	Blood test	162	2,3
	89.01.8	*Follow-up endocrinological visit*	Diagnosis and monitoring	Monitoring	159	2,2
**Total**					**7.179**	**100,0**

Table 3B. Top 10 most cancer-related prescription for Thyroid cancer (TC) by Anatomical Therapeutic Chemical (ATC), description, category in Drug Pharmacy database (DP)/Hospital Drugs database (HD) and phase of care. Epicost-2 prevalence population of 12,157 TC patients.

Database	Phase of care	ATC	Description	Category	N	%
HD, DP	**Initial**	H03AA01	*Levothyroxine sodium*	Thyroid hormones	11.764	51,9
HD		A11CC05	*Colecalciferol*	Support therapy	4.881	21,5

HD		A11CC04	*Calcitriol*	Support therapy	1.715	7,6
DP		B01AB05	*Enoxaparin*	Antithrombotics	725	3,2
HD		A12AX	*Calcium, combinations with vitamin D and/or other drugs*	Support therapy	672	3,0
HD, DP		A12AA20	*Calcium (different salts in combination)*	Support therapy	610	2,7
DP		V04CJ01	*Thyrotropin*	Support therapy	478	2,1
HD		H03AA02	*Oiothyronine sodium*	Thyroid hormones	445	2,0
HD		A12AA04	*Calcium carbonate*	Support therapy	426	1,9
DP		H02AB07	*Prednisone*	Support therapy	198	0,9
	**Total**				**22.656**	**100,0**
DP	**Continuing**	H03AA01	*Levothyroxine sodium*	Thyroid hormones	198.300	58,6
DP		A11CC05	*Colecalciferol*	Support therapy	63.629	18,8
DP		A11CC04	*Calcitriol*	Support therapy	32.312	9,6
HD, DP		A12AX	*Calcium, combinations with vitamin D and/or other drugs*	Support therapy	10.588	3,1
DP		A12AA04	*Calcium carbonate*	Support therapy	8.501	2,5
DP		A12AA20	*Calcium (different salts in combination)*	Support therapy	5.981	1,8
DP		H03AA02	*Liothyronine sodium*	Thyroid hormones	5.143	1,5
DP		B01AB05	*Enoxaparin*	Antithrombotics	4.654	1,4
HD, DP		V04CJ01	*Thyrotropin*	Support therapy	3.105	0,9
HD, DP		A11CC06	*Calcifediol*	Support therapy	2.242	0,7
	**Total**				**338.113**	**100,0**
HD, DP	**Final**	H03AA01	*Levothyroxine sodium*	Thyroid hormones	1.689	51,1
HD		A11CC05	*Colecalciferol*	Support therapy	403	12,2
HD, DP		A11CC04	*Calcitriol*	Support therapy	373	11,3
DP		B01AB05	*Enoxaparin*	Antithrombotics	303	9,2
HD		A12AX	*Calcium, combinations with vitamin D and/or other drugs*	Support therapy	152	4,6
HD, DP		A12AA20	*Calcium (different salts in combination)*	Support therapy	108	3,3
DP		L01XE12	*Vandenatib*	Chemotherapy	85	2,6
HD		H03AA02	*Liothyronine sodium*	Thyroid hormones	71	2,1
HD		A12AA04	*Calcium carbonate*	Support therapy	47	1,4
DP		V04CJ01	*Thyrotropin*	Support therapy	18	0,5
	**Total**				**3.308**	**100,0**

Table 4B. Top 10 most frequent cancer-related procedures for Skin Melanoma by International statistical classification of disease (ICD9-CM), description, category, subcategory in Hospital Admissions and Discharge databases (HA) and phase of care. Epicost-2 prevalence population of 4,176 SM patients.

Phase of care	ICD9-CM code	Description	Category	Subcategory	N	%
**Initial**	86,4	*Radical excision of skin lesion*	Surgery	Surgery	1.150	26,5
	40,23	*Excision of axillary lymph node*	Surgery	Lymphadenectomy	458	10,5
	86,3	*Other local excision or destruction of lesion or tissue of skin and subcutaneous tissue*	Surgery	Plastic Surgery	228	5,2

	40,24	*Excision of inguinal lymph node*	Surgery	Lymphadenectomy	224	5,2
	99,29	*Injection or infusion of other therapeutic or prophylactic substance*	Support therapy	Support therapy	174	4,0
	89,52	*Electrocardiogram*	Diagnosis and monitoring	Cardiologic assessment	155	3,6
	86,59	*Closure of skin and subcutaneous tissue of other sites*	Surgery	Other surgery	140	3,2
	92,16	*Scan of lymphatic system*	Diagnosis and monitoring	High diagnostic	130	3,0
	87,44	*Routine chest x-ray, so described*	Diagnosis and monitoring	Conventional radiology	116	2,7
	40,51	*Radical excision of axillary lymph nodes*	Surgery	Lymphadenectomy	82	1,9
**Total**					**4.344**	**100,0**
**Continuing**	86.4	*Radical excision of skin lesion*	Surgery	Surgery	3.589	22,5
	40,23	*Excision of axillary lymph node*	Surgery	Lymphadenectomy	1.246	7,8
	40,24	*Excision of inguinal lymph node*	Surgery	Lymphadenectomy	679	4,3
	86,3	*Other local excision or destruction of lesion or tissue of skin and subcutaneous tissue*	Surgery	Plastic Surgery	614	3,8
	89,52	*Electrocardiogram*	Diagnosis and monitoring	Cardiologic assessment	604	3,8
	87,44	*Routine chest x-ray, so described*	Diagnosis and monitoring	Conventional radiology	561	3,5
	99,29	*Injection or infusion of other therapeutic or prophylactic substance*	Support therapy	Support therapy	538	3,4
	86,59	*Closure of skin and subcutaneous tissue of other sites*	Surgery	Other surgery	484	3,0
	88,72	*Diagnostic ultrasound of heart*	Diagnosis and monitoring	Cardiologic assessment	449	2,8
	92,16	*Scan of lymphatic system*	Diagnosis and monitoring	High diagnostic	376	2,4
**Total**					**15.963**	**100,0**
**Final**	86,4	*Radical excision of skin lesion*	Surgery	Surgery	158	9,1
	87,44	*Routine chest x-ray, so described*	Diagnosis and monitoring	Conventional radiology	122	7,1
	89,52	*Electrocardiogram*	Diagnosis and monitoring	Cardiologic assessment	111	6,4
	99,21	*Injection of antibiotic*	Support therapy	Support therapy	104	6,0
	87,03	*Computerized axial tomography of head*	Diagnosis and monitoring	High diagnostic	100	5,8
	99,29	*Injection or infusion of other therapeutic or prophylactic substance*	Support therapy	Support therapy	97	5,6

	88,76	*Diagnostic ultrasound of abdomen and retroperitoneum*	Diagnosis and monitoring	Ultrasonography	64	3,7
	99,28	*Injection or infusion of biological response modifier [BRM] as an antineoplastic agent*	Biologic therapy	Biologic therapy	60	3,5
	88,72	*Diagnostic ultrasound of heart*	Diagnosis and monitoring	Cardiologic assessment	56	3,2
	40,23	*Excision of axillary lymph node*	Surgery	Lymphadenectomy	53	3,1
**Total**					**1.727**	**100,0**

Table 5B. Top 10 most frequent cancer-related procedures for Skin Melanoma by International statistical classification of disease (ICD9-CM), description, category, subcategory in Outpatients Services database (OPS) and phase of care. Epicost-2 prevalence population of 4,176 SM patients.

Phase of care	ICD9-CM code	Description	Category	Subcategory	N	%
**Initial**	89,13	*Neurologic examination*	Diagnosis and monitoring	Specialist examination	215	14,0
	90.05.5	*Alpha 1 fetoprotein [S/La/Alb]*	Diagnosis and monitoring	Bio-marker	162	10,6
	90.55.1	*Cancer Antigen 125*	Diagnosis and monitoring	Genetic marker	138	9,0

	89.7B.9	*FIRST ENT VISIT. Included, based on the specific clinical problem: possible otomicroscopy, vestibular function examination, use of optical fibers, removal of earwax*	Diagnosis and monitoring	Specialist examination	88	5,7
	88.77.2	*Diagnostic ultrasound of peripheral vascular system*	Diagnosis and monitoring	Ultrasonography	74	4,8
	90.93.3	*Microscopic examination of specimen from lower gastrointestinal tract and of stool, culture and sensitivity*	Diagnosis and monitoring	Culture test	74	4,8
	93.39.2	*Massotherapy for lymphatic drainage*	Diagnosis and monitoring	Physiatry	58	3,8
	92.19.8	*Total body positron emission tomography (PET) with computerized Axial tomography (CAT)*	Diagnosis and monitoring	High diagnostics	53	3,5
	89.01.N	*Interview and evaluation, described as brief*	Diagnosis and monitoring	Specialist examination	47	3,1
	90.18.4	*Neuron specific enolasis*	Diagnosis and monitoring	Bio-marker	43	2,8
**Total**					**1.533**	**100,0**
**Continuing**	90.05.5	*Alpha 1 fetoprotein [S/La/Alb]*	Diagnosis and monitoring	Blood test	1.744	13,9
	89,13	*Neurologic examination*	Diagnosis and monitoring	Specialist examination	1.563	12,4
	90.55.1	*Cancer Antigen 125*	Diagnosis and monitoring	Genetic marker	1.496	11,9
	89.7B.9	*FIRST ENT VISIT. Included, based on the specific clinical problem: possible otomicroscopy, vestibular function examination, use of optical fibers, removal of earwax*	Diagnosis and monitoring	Specialist examination	692	5,5
	90.36.6	*Microscopic examination of specimen from ear, nose, throat, and larynx, cell block and Papanicolaou smear*	Diagnosis and monitoring	Blood test	688	5,5
	90.93.3	*Microscopic examination of specimen from lower gastrointestinal tract and of stool, culture and sensitivity*	Diagnosis and monitoring	Culture test	637	5,1
	88.77.2	*Diagnostic ultrasound of peripheral vascular system*	Diagnosis and monitoring	Ultrasonography	418	3,3
	93.39.2	*Massotherapy for lymphatic drainage*	Diagnosis and monitoring	Physiatry	348	2,8
	90.18.4	*Neuron specific enolasis*	Diagnosis and monitoring	Bio-marker	340	2,7
	89.7A.6	*First vascular surgicalvisit*	Diagnosis and monitoring	Specialist examination	335	2,7
**Total**					**12.576**	**100,0**
**Final**	90.36.6	*Microscopic examination of specimen from ear, nose, throat, and larynx, cell block and Papanicolaou smear*	Diagnosis and monitoring	Blood test	79	11,4
	89,13	*Neurologic examination*	Diagnosis and monitoring	Specialist examination	71	10,2
	92.29.6	*In vivo dosimetry*	Radiotherapy	Radiotherapy	63	9,1
	90.93.3	*Microscopic examination of specimen from lower gastrointestinal tract and of stool, culture and sensitivity*	Diagnosis and monitoring	Culture test	48	6,9
	88.90.2	*Three-dimensional computerized Axial tomography (CAT) reconstruction*	Diagnosis and monitoring	High diagnostics	47	6,8
	90.05.5	*Alpha 1 fetoprotein [S/La/Alb]*	Diagnosis and monitoring	Blood test	44	6,3

	90.55.1	*Cancer Antigen 125*	Diagnosis and monitoring	Genetic marker	37	5,3
	92.19.8	*Total body positron emission tomography (PET) with computerized Axial tomography (CAT)*	Diagnosis and monitoring	High diagnostics	36	5,2
	89.7B.9	*FIRST ENT VISIT. Included, based on the specific clinical problem: possible otomicroscopy, vestibular function examination, use of optical fibers, removal of earwax*	Diagnosis and monitoring	Specialist examination	29	4,2
	92.29.K	*Other radiotherapeutic procedure*	Radiotherapy	Radiotherapy	28	4,0
**Total**					**482**	**100,0**

Table 6B. Top 10 most frequent cancer-related prescription for Skin Melanoma by Anatomical Therapeutic Chemical (ATC), decsription, category in Drug Pharmacy database (DP)/Hospital Drugs database (HD) and phase of care. Epicost-2 prevalence population of 4,176 SM patients.

**Dataase**	**Phase of care**	**ATC**	**Description**	**Category**	**N**	**%**
HD	**Initial**	A02BC02	*Pantoprazole*	Support therapy	4.412	14,8
HD		B01AC06	*Acetylsalicylic acid*	Antithrombotics	3.936	13,2
DP		L03AB04	*Interferon alfa-2a*	Chemotherapic drug	2.655	8,9
HD		A02BC01	*Omeprazole*	Support therapy	2.416	8,1

HD, DP		B01AB05	*Enoxaparin*	Antithrombotics	1.965	6,6
HD, DP		J01CR02	*Amoxicillin and beta-lactamase inhibitor*	Antibiotics	1.912	6,4
DP		L03AB05	*Interferon alfa-2b*	Chemotherapic drug	1.049	3,5
HD		H02AB07	*Prednisone*	Cortisone	1.046	3,5
HD		S01ED51	*Timolol, combinations*	Glaucoma medication	1.000	3,4
HD		N03AX16	*Pregabalin*	Analgesics	767	2,6
	**Total**				**29.724**	**100,0**
HD, DP	**Continuing**	B01AB05	*Enoxaparin*	Antithrombotics	7.109	3,7
DP		L03AB04	*Interferon alfa-2a*	Chemotherapic drug	6.998	3,7
DP		L03AB05	*Interferon alfa-2b*	Chemotherapic drug	5.661	3,0
HD		A02BC02	*Pantoprazole*	Support therapy	4.412	2,3
HD		B01AC06	*Acetylsalicylic acid*	Antithrombotics	3.936	2,1
DP		B01AF01	*Rivaroxaban*	Antithrombotics	3.261	1,7
DP		B01AC04	*Clopidogrel*	Antithrombotics	2.970	1,6
HD		A02BC01	*Omeprazole*	Support therapy	2.416	1,3
HD		J01CR02	*Amoxicillin and beta-lactamase inhibitor*	Antibiotics	1.814	0,9
HD, DP		H02AB07	*Prednisone*	Cortisone	1.769	0,9
	**Total**				**191.405**	**100,0**
HD	**Final**	B01AB05	*Enoxaparin*	Antithrombotics	4.412	31,7
HD		L01XE23	*Dabrafenib*	Biologic therapy	3.936	28,3
HD		B01AC04	*Clopidogrel*	Antithrombotics	2.416	17,4
HD, DP		L01AX03	*Temozolomide*	Chemotherapic drug	1.871	13,5
HD		L01XE25	*Trametinib*	Biologic therapy	1.814	13,1
HD, DP		L03AB04	*Interferon alfa-2a*	Chemotherapic drug	1.215	8,7
HD		H02AB07	*Prednisone*	Cortisone	767	5,5
HD		L01XC17	*Nivolumab*	Biologic therapy	711	5,1
HD		B01AF01	*Rivaroxaban*	Antithrombotics	594	4,3
HD		B01AX05	*Fondaparinux*	Antithrombotics	534	3,8
	**Total**				**13.900**	**100,0**
